# Functional significance of U2AF1 S34F mutations in lung adenocarcinomas

**DOI:** 10.1038/s41467-019-13392-y

**Published:** 2019-12-13

**Authors:** Mohammad S. Esfahani, Luke J. Lee, Young-Jun Jeon, Ryan A. Flynn, Henning Stehr, Angela B. Hui, Noriko Ishisoko, Eric Kildebeck, Aaron M. Newman, Scott V. Bratman, Matthew H. Porteus, Howard Y. Chang, Ash A. Alizadeh, Maximilian Diehn

**Affiliations:** 1 Stanford Cancer Institute, Stanford University, Stanford, USA; 2 Division of Oncology, Department of Medicine, Stanford University, Stanford, USA; 3 Department of Radiation Oncology, Stanford University, Stanford, USA; 4 Department of Chemistry, Stanford University, Stanford, USA; 5 Department of Pathology, Stanford University, Stanford, CA USA; 6 Department of Bioengineering, Stanford University, Stanford, USA; 7 Department of Pediatrics, Stanford University, Stanford, USA; 8 Institute for Stem Cell Biology and Regenerative Medicine, Stanford University, Stanford, USA; 9 Department of Biomedical Data Science, Stanford University, Stanford, USA; 10 Howard Hughes Medical Institute, Stanford University, Stanford, CA USA; 11 Division of Hematology, Department of Medicine, Stanford University, Stanford, USA; 12 Present Address: Department of Radiation Oncology, University of Toronto, Toronto, CA USA

**Keywords:** Cancer, Cancer genetics, Lung cancer, Oncology

## Abstract

The functional role of U2AF1 mutations in lung adenocarcinomas (LUADs) remains incompletely understood. Here, we report a significant co-occurrence of U2AF1 S34F mutations with ROS1 translocations in LUADs. To characterize this interaction, we profiled effects of S34F on the transcriptome-wide distribution of RNA binding and alternative splicing in cells harboring the ROS1 translocation. Compared to its wild-type counterpart, U2AF1 S34F preferentially binds and modulates splicing of introns containing CAG trinucleotides at their 3′ splice junctions. The presence of S34F caused a shift in cross-linking at 3′ splice sites, which was significantly associated with alternative splicing of skipped exons. U2AF1 S34F induced expression of genes involved in the epithelial-mesenchymal transition (EMT) and increased tumor cell invasion. Finally, S34F increased splicing of the long over the short SLC34A2-ROS1 isoform, which was also associated with enhanced invasiveness. Taken together, our results suggest a mechanistic interaction between mutant U2AF1 and ROS1 in LUAD.

## Abstract (web summary)

The authors report a co-occurrence of the U2AF1 S34F splicing factor mutation and ROS1 translocations in lung adenocarcinomas and profile effects of S34F on transcriptome-wide RNA binding. They further show that U2AF1 S34F enhances invasive potential and alters splicing of ROS1 fusion transcripts

## Introduction

Splicing of precursor messenger RNAs (pre-mRNAs) is a critical step in the processing of gene transcripts encoding most eukaryotic proteins^1–3^, as part of an ordered set of reactions catalyzed by a large RNA-protein complex known as the spliceosome^4–6^. Alternative splicing serves to generate different mRNAs and proteins from a single transcript^7,8^ and plays critical roles in development, differentiation, and diverse human diseases including cancer^9–12^. One of the components of the spliceosome, the U2 Auxiliary Factor complex, consists of a small, 35 kDa subunit (U2AF1) and a large, 65 kDa subunit (U2AF2). As essential proteins, U2AF1 and U2AF2 are together required for binding of the U2 snRNP to the 3′ splice site (3′ SS) of most eukaryotic introns^13–17^.

Recently, somatic mutations in U2AF1 have been recurrently observed in several human neoplasms, including myelodysplastic syndrome (MDS), acute myeloid leukemia (AML), and lung cancers^18–24^. Mutations in U2AF1 were first discovered in MDS, clonal hematopoietic stem-cell disorders that are characterized by deregulated, dysplastic blood cell formation and cytopenias^19,21^. In both MDS and AML, U2AF1 mutations cluster in both zinc finger domains of the protein, resulting in corresponding hotspots at codon 34 (S34F and S34Y) and codon 157 (Q157R and Q157P)^19,21^. Furthermore, recurrent mutations have been found in multiple components of the RNA splicing machinery in a mutually exclusive manner in MDS/AML, targeting distinct components of this apparatus including U2AF1, SF3B1, SRSF2, and ZRSR2^24–29^. In the hematopoietic context, U2AF1 mutations alter splice-site recognition and pre-mRNA splicing in vivo^28,30–32^. Indeed, mutant U2AF1 results in significant changes in hematopoiesis in mouse models, including expansion of hematopoietic stem cells and associated cytopenias, as well as alternative splicing of genes recurrently mutated in MDS/AML^18^.

Separate studies also discovered recurrent U2AF1 mutations in lung cancers as part of large, genome-wide studies of somatic mutations in these tumors^20,33^. Surprisingly, while U2AF1 mutations have been observed in ~3% of lung adenocarcinomas (LUAD), their frequency in other lung cancer subtypes appears to be lower, including in squamous cell carcinomas (LUSC)^34,35^ and small cell carcinomas (SCLC)^36^. Interestingly, unlike hematological neoplasms including MDS where mutations targeting both zinc finger domains of U2AF1 are pervasive, S34F mutations targeting the first zinc finger domain are the most pervasive hotspot in lung cancers^37^. Finally, unlike in MDS/AML, U2AF1 S34Y variants are exceedingly rare in lung cancers, as are mutations in other components of the spliceosome. While this distinct pattern of somatic mutation and histological specificity in lung cancers suggests a functional interaction within these tumors, the role of S34F in LUADs remains incompletely understood. For example, while Brooks and colleagues identified commonly altered splicing events shared between lung cancers and AML harboring U2AF1 mutations^38^, it has been difficult to glean mechanistic insight about differences between these tumors from such studies. Separately, Fei and colleagues found that lung tumors harboring U2AF1 S34F invariably retain a wild-type allele of the gene and are critically dependent on this wild-type allele for cell survival. However, knocking out the S34F allele did not have an effect on cell death in vitro^39^. Therefore, the functional significance of U2AF1 mutations in lung cancer remains largely unknown.

Here, we confirm the specificity of S34F mutations in primary LUADs and demonstrate a significant co-occurrence of U2AF1 S34F mutations with ROS1 translocations in these tumors. To characterize the potential interactions of these two mutations we employed individual nucleotide resolution crosslinking and immunoprecipitation (iCLIP)-sequencing of RNA precursors to study the binding of wild-type and mutant U2AF1 at nucleotide resolution in LUAD cells harboring the SLC34A2-ROS1 fusion^40,41^. When compared to its wild-type counterpart, we found that U2AF1 S34F preferentially binds and modulates splicing of introns containing CAG trinucleotides at their 3′ splice junctions. We also show that overexpression of U2AF1 S34F leads to elevated expression of genes associated with the epithelial-mesenchymal transition (EMT). Consistent with this observation, U2AF1 S34F induces cell invasiveness and that this appears to be in part mediated via preferential splicing of the SLC34A2-ROS1 long isoform, whose expression also increases tumor cell invasion. Our results suggest a mechanistic interaction between mutant U2AF1 and ROS1 in LUADs that may be of clinical and therapeutic relevance.

## Results

### Co-occurrence of U2AF1 S34F and ROS1 translocations in LUAD

**Fig. 1:**
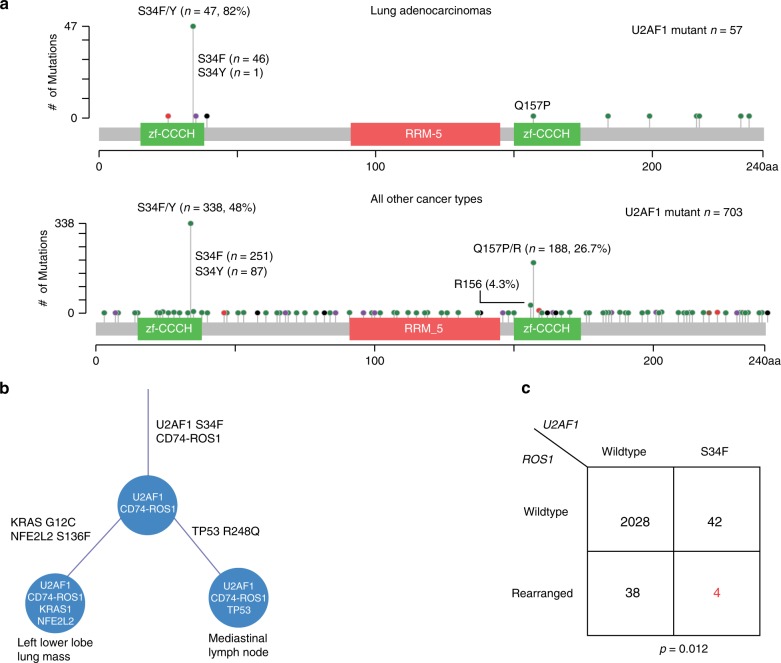
Recurrence and co-association of U2AF1 S34F mutations. **a** Recurrence of U2AF1 S34F mutations in multiple data sets including data summarized from cBioPortal, TRACERx, Stanford patients, and cancer cell lines including LUAD. **b** The approximate phylogeny tree of the two samples collected from a Stanford LUAD patient, illustrating U2AF1 S34F mutation and CD74-ROS1 fusion as truncal events. **c** Co-occurrence of SLC34A2-ROS1 fusions and U2AF1 S34F mutations in LUAD subset of cohort in **a**: A total of 2112 cases were analyzed, with highlighted cases of S34F mutations and ROS1 fusions shown in red. A Fisher’s exact test was performed to determine statistical significance.

To characterize the role of U2AF1 mutations, we collated sequencing data from a diverse set of 2112 LUADs, including cases from the original study identifying U2AF1 in LUAD^20^, The Cancer Genome Atlas^33^, metastatic tumors profiled with MSK-IMPACT^42^, tumors profiled as part of the TRACERx study^43^, and cases profiled using Stanford’s Solid Tumor Actionable Mutation Panel (STAMP)^44^. Within this relatively large group of LUADs, we identified 57 cases (2.7%) harboring U2AF1 mutations. Analysis of the distribution of these U2AF1 mutations across the protein revealed a single, dominant hotspot at S34F that comprised the majority of mutations in lung cancers (81%; Fig. 1a). This pattern differed from that observed in a set of 703 U2AF1 mutant tumors from other tumor histologies (Binomial test, *P* = 2.5E-7; Fig. 1a), where two hotspots were observed, one at codon S34 (48% of mutations) and another at Q157 (27% of mutations).

We next explored whether U2AF1 mutations were truncal events in LUADs analyzed by multi-region sequencing. Among three LUAD cases with U2AF1 mutations where more than one tumor region was profiled in the TRACERx study, the mutation was invariably present in all sequenced regions. We genotyped two tumor deposits from distinct anatomic sites in a patient with LUAD carrying both a U2AF1 S34F mutation and a ROS1 fusion. Interestingly, both tumor deposits contained the U2AF1 S34F mutation and CD74-ROS1 translocation, suggesting that these two events were most likely truncal (Fig. 1b; Supplementary Data 1a). One deposit also contained KRAS G12C and NFE2L2 S136F mutations and the other a TP53 R248Q mutation whose allele frequencies were all close to that of U2AF1 (~20%). We were struck by the presence of both U2AF1 S34F and a ROS1 translocation in the same tumor since we had observed two samples with U2AF1 S34F mutations and ROS1 translocations in a prior study^45^. To further investigate this connection, we examined the association between U2AF1 S34F mutations and ROS1 fusions in the above 2112 LUADs and found them to be significantly co-associated, with ~10% of tumors with either ROS1 translocations or U2AF1 S34F mutations also harboring the other mutation (overall prevalence for double mutant of 0.2%, Fisher’s exact test *P* = 0.012; Fig. 1c; Supplementary Data 2). This observation suggests a potential functional link between the two mutations in LUADs.

### Assessment of RNAs bound by wild-type and S34F mutant U2AF1

**Fig. 2:**
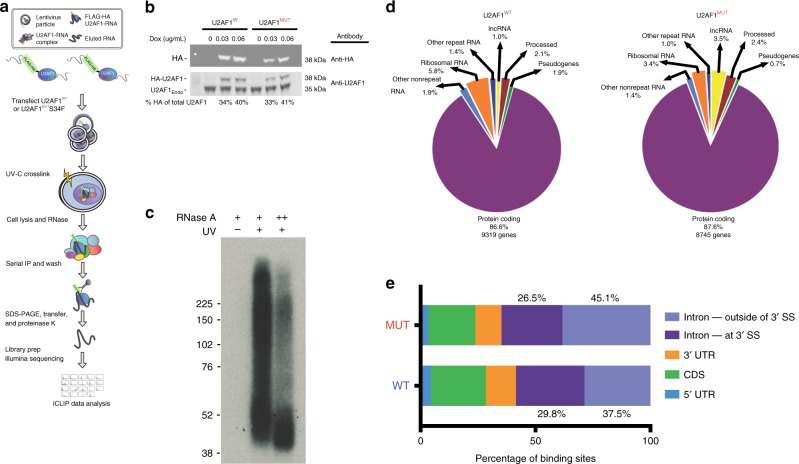
Genome-wide mapping of RNA interactions for wild-type and S34F mutant U2AF1. **a** Schematic illustration of U2AF1 iCLIP. IP, immunoprecipitation; RNase, ribonuclease. Doxycycline-inducible FLAG-HA-tagged wild-type and S34F-mutant U2AF1 plasmids were transfected into HCC78 cells. Induced lysates were purified on anti-Flag-M2 agarose beads followed by a series of wash steps to specifically elute FLAG peptide-containing complexes and recaptured with anti-HA agarose. Standard iCLIP steps were subsequently performed to generate deep sequencing libraries. **b** Western blots were performed to confirm doxycycline induction of FLAG-HA-U2AF1 wild-type and S34F mutant constructs utilizing anti-HA and anti-U2AF1 monoclonal antibodies. Percent of HA-tagged U2AF1 of total U2AF1 is shown. **c** Autoradiogram of ^32^P-labeled RNA crosslinked to Flag-HA-U2AF1 trimmed with two different concentrations of RNase A. RNA-protein complexes are seen in the purifications from Flag-HA-U2AF1 cells but not from the parental control cell line, HCC78. **d** Genomic distribution of U2AF1 iCLIP reads. lncRNA, long noncoding RNA. Wild-type and mutant U2AF1 iCLIP reads were annotated to known repetitive and non-repetitive regions of the human genome with percentage of total iCLIP reads shown. **e** Binding distribution of wild-type and S34F mutant U2AF1 iCLIP reads. *UTR* untranslated region, *CDS* coding sequence; 3′ SS.

To explore the role of U2AF1 S34F in LUAD harboring ROS1 translocations, we next sought to globally characterize RNA transcripts directly bound by the protein in cells carrying both somatic aberrations. To do so, we employed individual nucleotide resolution crosslinking and immunoprecipitation (iCLIP) profiling, allowing single-base resolution of interactions between RNA-binding proteins and RNAs in a transcriptome-wide manner (Fig. 2a)^46–49^. We selected HCC78 LUAD cells for these experiments since they fortuitously contain both the U2AF1 S34F mutation and an SLC34A2-ROS1 fusion (Supplementary Fig. 1A, B). This allowed us to study the role of U2AF1 in the presence of a ROS1 fusion. We initially attempted to generate U2AF1 wild-type or S34F homozygous HCC78 sub-clones utilizing transcription activator-like effector nucleases (TALENs) with U2AF1 wild-type or S34F donor vectors. However, all resulting single cell clones were heterozygous, with each construct preferentially replacing its endogenous allele and retaining at least one wild-type copy of U2AF1 (Methods; Supplementary Fig. 1C). This finding suggests a dependence of HCC78 cells on the presence of one wild-type and one mutant copy of U2AF1 and confirms a recently published study^39^.

Given this finding, we instead chose to express epitope tagged versions of U2AF1 in HCC78 cells. Specifically, we introduced doxycycline-inducible versions of U2AF1 into HCC78 cells using plasmid constructs encoding either wild-type or S34F mutant isoforms, each being dually tagged with FLAG and hemagglutinin (HA) epitope tags to facilitate efficient serial purification. Doxycycline was titrated to achieve expression of each of the tagged isoforms at near endogenous levels (Fig. 2b). Following serial affinity purification of immunoprecipitated complexes, evaluation of UV-crosslinked RNAs by autoradiography demonstrated successful recovery of RNA protected and footprinted by U2AF1 (Fig. 2c). Deep sequencing of these immunoprecipitated RNAs revealed the preferential binding of U2AF1 to protein-coding mRNAs (~87%) compared to non-coding RNAs (13%), and this preference was unchanged in the presence of S34F (Fig. 2d, Supplementary Data 3). We validated iCLIP sequencing results for two randomly selected transcripts that demonstrated differential binding by these two U2AF1 isoforms using RNA immunoprecipitation followed by quantitative PCR (RIP-qPCR; Supplementary Fig. 1D).

### S34F shifts U2AF1 cross-linking at intronic 3′ splice sites

**Fig. 3:**
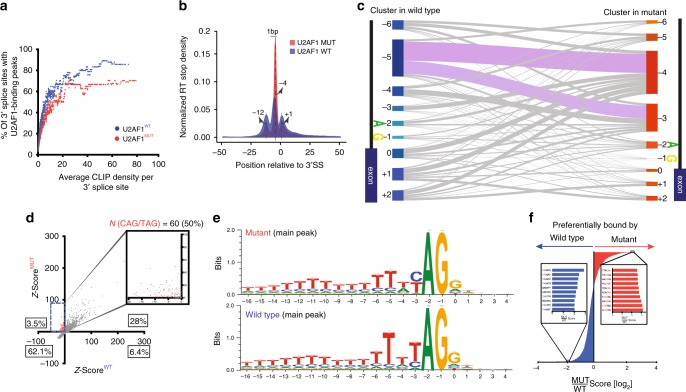
Determining binding specificities of wild-type and mutant U2AF1. **a** U2AF1 binds a subset of 3’ SSs. Maximum-likelihood analysis was utilized to determine the 3′ SS occupancy of wild-type and S34F mutant U2AF1. Each dot represents an average occupancy of a group of 40 genes, in relation to average CLIP density per 3′ SS. **b** Metagene analysis of wild-type and S34F mutant U2AF1 binding interactions to pre-mRNA 3′ SSs. Normalized RT-stop density is shown across 3′ SS positions on the *x*-axis. **c** RT stops >10 were assigned to clusters defined from a window of 3′ SSs of −6 to + 2 nucleotides utilizing 3′ SS annotation files. The cluster assignments for wild-type and mutant samples are shown in left and right, respectively. **d** Z-scores were generated based on random hexamer nucleotide motif frequencies bound to wild-type and S34F mutant U2AF1. A subset of Z-scores showing enrichment of 50% of the hexamers bound to mutant U2AF1 for CAG and its reverse complement CTG trinucleotides. **e** WebLogos depicting binding preferences of wild-type and mutant U2AF1 for the main peaks in **b**. Position 0 is the intron exon junction depicted by the figure. **f** Generation of a preferential binding score to wild-type and mutant U2AF1. The top 10 scoring hexamers and its common trinucleotide sequences preferentially bound to mutant U2AF1 and wild-type U2AF1 are shown in red and blue, respectively.

To begin to explore the iCLIP data, we evaluated the specific mRNA regions bound by U2AF1. As expected, the majority of mRNA binding sites for U2AF1 were within introns and this was similar for both isoforms (Fig. 2e). We next more closely examined the specific regions within introns preferentially bound by U2AF1, initially focusing on their tendency to occupy 3′ splice sites. We used a saturation analysis to compare U2AF1 isoforms for their binding to these intronic regions and observed a saturation plateau for binding, (Fig. 3a, Methods), consistent with prior findings for U2AF2^50^. However, at the CLIP density where this saturation was observed, wild-type U2AF1 occupied ~86% of 3′ splice sites while its S34F mutant counterpart occupied ~70% of corresponding regions (Fig. 3a). This difference suggests a moderate reduction in the preference of S34F mutant U2AF1 for 3′ splice-site binding when compared to its wild-type counterpart.

Consistent with U2AF1’s role in facilitating binding of U2 snRNPs to the AG splice acceptor dinucleotide at the 3′ end of introns, we found strong enrichment of iCLIP cross-linking sites at this location for both wild-type and S34F mutant isoforms (Fig. 3b). For wild-type U2AF1, the most frequently crosslinked nucleotide was the A of the dinucleotide, with two smaller flanking peaks at −12 and + 1. However, for S34F U2AF1 the most frequently cross-linked nucleotide was shifted by 1 position to the G nucleotide (Fig. 3b, c). The two peaks at −12 and + 1 were preserved but of lower relative amplitude in the mutant, suggesting a more focused interaction closer to the 3′ SS.

We next compared binding of the two isoforms at the level of individual introns. We clustered individual intronic regions based on their binding patterns, allowing us to directly compare the positions cross-linked by wild-type versus S34F mutant U2AF1 at each 3′SS. This analysis also revealed strongest cross-linking by both U2AF1 isoforms focused at the terminal five nucleotides of each intron, supported by the largest clusters of binding mapping to these positions. Within this higher resolution analysis, we confirmed the average results summarized above across all introns differentially bound by the two U2AF1 isoforms, again observing a single nucleotide shift between them. Specifically, the largest cluster of individual introns were cross-linked by wild-type U2AF1 at position −5, but in these same introns, the S34F mutant primarily cross-linked at position −4 (Supplementary Fig. 2A, Fig. 3c, Methods). Collectively, these results suggest a moderate decrease in cross-linking of introns by the S34F mutant isoform and an associated single nucleotide shift in its cross-linking. This could reflect structural constraints for cross-linking inherent to substitution of serine by phenylalanine at codon 34. Alternatively, this qualitative change could reflect a decrease in binding of introns by the S34F mutant isoform.

### U2AF1 S34F preferentially binds *CAG* sequences at 3′ SSs

To further determine the binding specificity of wild-type and S34F mutant isoforms at the 3′ SS, we examined hexamer nucleotide motifs surrounding individual U2AF1-crosslinked RNA nucleotides (Fig. 3d, Methods). While greater than 90% of all hexamer sequences had similar frequencies, 3.5% were selectively enriched among binding sites preferred by the S34F mutant. Among these sites, we observed a striking enrichment of *CAG* (and its reverse complement *CTG*), which was contained in 50% of the corresponding sequences. We observed a preference in binding for *CAG* over *TAG* trinucleotides in the S34F mutant compared to the wild-type (Fig. 3e). Moreover, when we examined the two smaller flanking peaks at positions −12 and + 1 we also observed a similar enrichment for *CAG* over *TAG*, suggesting U2AF1 also contacts transcripts at these positions (Supplementary Fig. 3; Methods). Similarly, when ranking hexamers by their relative binding frequency by mutant versus wild-type U2AF1, the majority of sequences most preferentially bound by the S34F mutant contained *CAG* trinucleotides (Fig. 3f, Supplementary Fig. 4). Conversely, we observed a preference for the *TAG* trinucleotide in hexamers preferentially bound by wild-type U2AF1. Taken together, these data indicate that S34F mutant U2AF1 increases the frequency of binding *CAG* over *TAG* 3′ SSs compared to wild-type.

### Impact of U2AF1 S34F on RNA binding and gene expression

**Fig. 4:**
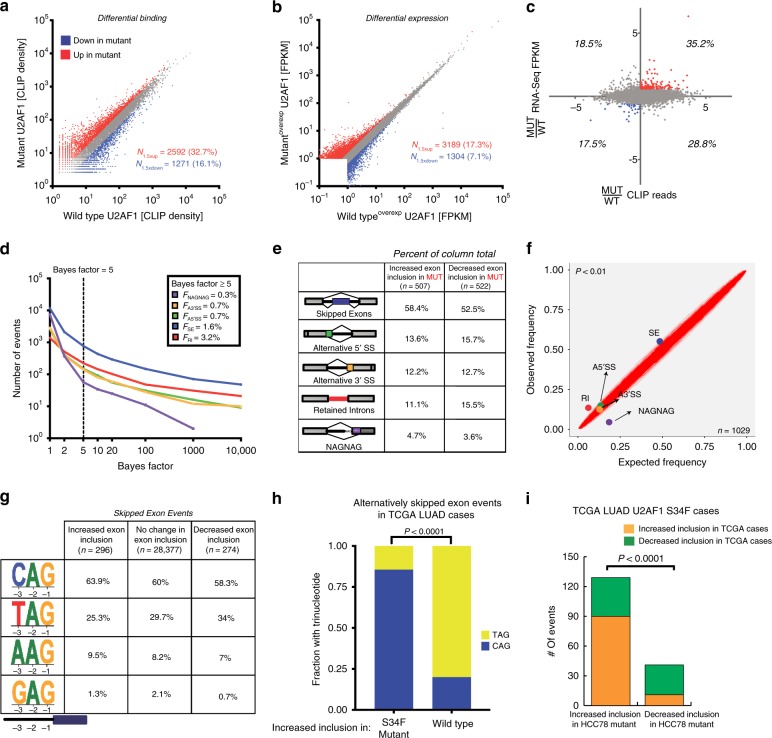
Global binding and expression differences in wild-type and mutant U2AF1. **a** U2AF1 S34F globally alters binding in HCC78. Scatter plots comparing CLIP reads of individual genes bound to wild-type and mutant U2AF1. Genes that were preferentially bound (fold change >1.5) in favor of mutant (red) and wild-type (blue) are shown. **b** U2AF1 S34F globally alters expression in HCC78. Scatter plots comparing FPKM values of individual genes bound to wild-type and mutant U2AF1. Genes that were preferentially expressed (fold change >1.5) in favor of mutant (red) and wild-type (blue) are shown. **c** Comparison of mutant to wild-type ratios U2AF1 CLIP reads and FPKM values of individual genes. 87 genes for mutant and 23 genes for wild-type are shown to be differentially bound and expressed in favor of the mutant and wild-type, respectively. **d** Cumulative frequencies of alternative splicing events. A statistically significant threshold for alternative splicing events comparing wild-type and mutant U2AF1 (Bayes factor >5) is shown. A3′SS, alternative 3′ SSs; A5’SS, alternative 5’ splice sites; SE, skipped exons; RI, retained introns. **e** Alternative splicing events preferentially spliced in favor of mutant or wild-type U2AF1 at greater than 10% frequency (Bayes factor >5). ∆PSI = difference in “percentage spliced in”. **f** Scatter plot comparison of expected alternative splicing events and observed events as a percentage of all events. Gray area represents statistically significant region with *P* < 0.01. **g** The frequency of each trinucleotide at 3′ splice sites of alternatively skipped exons in U2AF1 S34F mutant and wild-type transduced cells based on their change in inclusion. **h** Fraction of skipped exon events with TAG or CAG at the 3′ splice site in wild-type and U2AF1 S34F mutant TCGA LUAD cases (*n* = 175 for wild-type and 125 for mutant). **i** Overlap of differentially skipped exon events in HCC78 (Z-score of ψ greater/less than ±1.64, *P* = 0.05) and TCGA U2AF1 S34F mutant LUAD cases (*P* = 0.05).

To further characterize the global effects of U2AF1 and its two isoforms across the transcriptome, we compared their preference for binding to individual genes (Fig. 4a) and corresponding changes in gene expression (Fig. 4b). When comparing the number of genes differentially bound by the two U2AF1 isoforms (≥1.5-fold change; Fig. 4a), nearly a third of bound genes were preferentially bound by S34F mutant U2AF1 (32.7%), as compared with about a sixth preferentially bound by its wild-type counterpart (16.1%). For comparison, using the same thresholds, 17.1% of differentially expressed genes were more highly expressed in the mutant transduced, versus 7.1% more highly expressed in the wild-type transduced cells (Fig. 4b, Supplementary Data 4, Methods). However, when integrating these results, only a minority of genes differentially bound by S34F mutant versus wild-type U2AF1 were also differentially expressed (Fig. 4c). Indeed, across the transcriptome differential binding by U2AF1 isoforms was not significantly correlated with differential expression (*R*^*2*^ = *0.06*, *P* = *N.S*.) (Fig. 4c). Therefore, U2AF1 S34F results in a larger effect on RNA binding than on RNA expression, with no significant direct relationship between these effects at a transcriptome-wide level.

### Impact of U2AF1 S34F on alternative mRNA splicing

We next examined the transcriptome-wide effects of U2AF1 S34F on splicing. To do so we employed Mixture of Isoforms (MISO) analysis to compare alternative splicing events (Supplementary Data 5, Methods)^51^. The most common type of alternative splicing events that differed when overexpressing the two isoforms were skipped exons, with 274 exons being preferentially skipped in S34F transduced cells and 296 being preferentially skipped in wild-type U2AF1 transduced cells (Fig. 4d, e). The frequencies of skipped exons and retained introns were significantly increased while NAGNAG events were significantly decreased when comparing mutant to wild-type isoform transduced cells (Chi-squared test, *P* < 0.01; Fig. 4f).

To more deeply explore these alternative splicing results, we next more closely examined differentially skipped exons. Consistent with the binding affinities we observed above, *CAG* trinucleotides were more frequent and *TAG* trinucleotides were less frequent at 3′ splice junctions preceding preferentially included exons in S34F transduced compared to wild-type transduced cells (Fig. 4g). To confirm this result in primary LUAD samples, we analyzed TCGA LUAD cases (Methods) and observed a significant preference of CAG over TAG at the 3′ splice junctions preceding differentially skipped exons in U2AF1 S34F mutant cases (Fisher’s exact test, *P* < 0.0001; Fig. 4h), which is consistent with previous finding from a smaller set of LUAD cases^38^. Finally, we wondered whether alternative splicing events we observed in HCC78 cells were recapitulated in primary LUAD samples. We therefore evaluated the co-association of increased or decreased exon inclusion in HCC78 with that observed in U2AF1 S34F mutant TCGA LUAD cases (Methods). Interestingly, we found a significant overlap of skipped exon events in the two data sets (Fisher’s exact test, *P* < 0.001; Fig. 4i), suggesting that HCC78 cells recapitulate alternative splicing seen in primary LUAD. Taken together, these results suggest a model in which the differential binding preferences of wild-type and S34F mutant U2AF1 at 3′ splice junctions lead to differences in alternative splicing.

### Association of altered U2AF1 binding with mRNA splicing

To further explore the potential role of U2AF1 RNA binding in alterative splicing, we examined the relationship between binding of introns by U2AF1 isoforms in our iCLIP data with corresponding effects on alternative splicing within the RNA-Seq data. We observed that the differential splicing events involving skipped exons with large changes in prevalence between cells transduced with the two U2AF1 isoforms were also highly enriched for having adjacent introns that were differentially bound by the corresponding isoforms (Fisher’s exact test, *P* = 0.003; Supplementary Fig. 2B). Of interest, this association between differential binding and differential splicing of individual introns by U2AF1 isoforms did not extend to retained introns or NAGNAG introns (Fisher’s exact test, *P* = 0.83 and *P* = 0.74, respectively), suggesting the mechanisms of differential splicing induced by the presence of U2AF1 S34F may differ depending on the type of alternative splicing event.

### U2AF1 S34F increases EMT gene expression and invasiveness

**Fig. 5:**
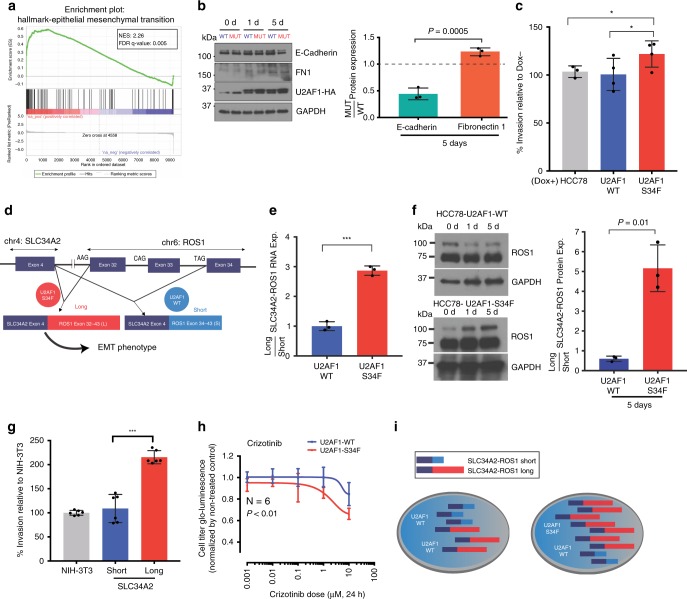
Functional significance of U2AF1 S34F in relation to SLC34A2-ROS1 fusions. **a** Gene set enrichment analysis (GSEA) plot of differential expression data. The most enriched gene set is the hallmark epithelial mesenchymal transition. **b** Relative protein expression of E-cadherin and fibronectin 1 (FN1) in HCC78 cells. Experiments were performed in triplicate. **c** Overexpression of U2AF1 S34F mutant increases invasion potential in HCC78. Wild-type and mutant U2AF1 were overexpressed in a doxycycline-inducible manner and plated for 48 h. **d** Schematic of SLC34A2-ROS1 splicing in HCC78. Exon 4 of SLC34A2 is fused to exon 32 or exon 34 of ROS1, creating long and short isoforms, respectively. **e** Relative expression of long:short SLC34A2-ROS1 isoform ratios in mutant and wild-type U2AF1 overexpressed cell lines as measured by RT-qPCR using isoform specific primers. The long and short isoforms in the mutant and wild-type overexpressed U2AF1 cell lines were normalized to the parental cell line, HCC78. **f** Relative protein expression of long to short SLC34A2-ROS1 isoforms in mutant and wild-type U2AF1 transduced cell lines as measured by Western blots, where HCC78 isogenic cells were exposed by 0.2 uµg/ml of doxicycline for 0, 1 day and 5 day to induce the U2AF1 expression. The long and short isoforms in the mutant and wild-type transduced U2AF1 cell lines were normalized to the GAPDH, with densitometry data from 5d summarized in bar plot (right). The band intensity was measured by Image J software. **g** The long ROS1 isoform increases invasion potential in the NIH-3T3 cell line after 48 h. The short and long SLC34A2-ROS1 isoforms were transduced in NIH-3T3 fibroblasts using lentivirus. **h** Sensitivity of wild-type versus S34F mutant U2AF1 transduced HCC78 cells to the ROS1 inhibitor crizotinib. Experiments were done with 6 technical replicates where cells were exposed to the drug for 24 h and cell survival rate was measured by manufacturer’s standard protocol (CellTiter-Glo® Luminescent Cell Viability Assay, Promega). **i** Proposed mechanism of U2AF1 S34F in LUADs with ROS1 fusions. U2AF1 S34F alters short and long SLC34A2-ROS1 isoform ratios. Error bars are used to illustrate the standard deviations around the means.

To glean potential insights into the functional role of U2AF1 S34F in LUADs, we next examined the transcriptome-wide changes induced by its expression in the drug-inducible isogenic HCC78 lines described above. We found that genes preferentially induced by S34F mutant U2AF1 were significantly enriched for those associated with epithelial to mesenchymal transition (EMT)^44^ (Gene Set Enrichment Analysis NES = 2.26, FDR Q-value = 0.005; Fig. 5a) and confirmed this finding for 10 EMT-associated genes, including the canonical markers *ZEB1* and *VIM* (Supplementary Fig. 5A). Alternative splicing of a subset of canonical EMT genes was affected by U2AF1 S34F (Supplementary Data 6) and the fold change in gene expression of EMT genes was statistically significantly correlated with U2AF1 cross-liking, suggesting that for *EMT* genes there is an association between expression and U2AF1 cross-linking (Spearman *r* = 0.52, *P* = 0.05; Supplementary Fig. 5B). Consistent with the gene expression results, protein expression of FN1 increased and E-cadherin decreased in S34F U2AF1-transduced cell (Fig. 5b). To test if these expression changes resulted in functional impacts, we next examined in vitro invasion of the transduced cells. Consistent with the gene expression changes, we observed a statistically significant increase in relative invasiveness induced by doxycycline-induced overexpression of S34F mutant compared to wild-type U2AF1 (Fig. 5c). Importantly, this difference in invasive potential was not associated with a significantly higher proliferative rate (Supplementary Fig. 6A). Taken together, these observations suggest a mechanism for U2AF1 S34F in altering LUAD phenotypes by inducing a gene expression program associated with EMT.

### U2AF1 S34F alters ratio of SLC34A2-ROS1 isoforms in HCC78

Having observed induction of an EMT program and associated invasiveness of HCC78 cells by S34F mutant U2AF1, we tested whether a similar effect was seen in LUADs in TCGA harboring the same mutation. However, we could not confirm a significant association between U2AF1 S34F mutation and the induction of an EMT program in corresponding RNA-Seq data (data not shown). Since only one of the 11 TCGA tumors harboring U2AF1 S34F were known to also harbor a ROS1 fusion as seen in HCC78 cells, we hypothesized that the induction of this program might be unique to tumors harboring both lesions, with the effect mediated by ROS1 splicing of the long and short isoforms.

Accordingly, we studied the effect of expression of S34F mutant U2AF1 on alternative splicing of the ROS1 fusion found in HCC78 cells^52^. The SLC34A2-ROS1 fusion gives rise to two major protein isoforms by alternative splicing: a long isoform that includes exons 32–43 of ROS1 and a short isoform that includes only exons 34–43 (Supplementary Fig. 1B and Fig. 5d)^50,53–55^. The 3′ splice site sequence immediately upstream of exon 32 is AAG and of exon 34 is TAG. Intriguingly, the frequencies of observed trinucleotide binding for S34F versus wild-type U2AF1 suggest a >2 fold preference of the mutant for AAG, thus potentially favoring splicing of the long isoform (Supplementary Fig. 2). We therefore asked whether overexpression of U2AF1 S34F affects the relative proportion of the two isoforms. Consistent with our prediction from binding preferences, we detected a significant relative increase (~3-fold) in the expression of the long SLC34A2-ROS1 isoform induced by S34F mutant but not wild-type U2AF1 (Fig. 5e), and confirmed these results using a previously published data set^39^ (Supplementary Fig. 7). Knockdown of total U2AF1 using siRNA also perturbed ROS1 splicing in HCC78 cells, resulting in a ~2-fold relative increase in expression of the short ROS1 fusion isoform (Supplementary Fig. 6B). We then compared protein levels of long and short SLC34A2-ROS1 isoforms in HCC78 and found a significant enrichment of the long isoform in HCC78 cells over-expressing U2AF1-S34F (Student’s *t*-test, *P* = 0.01; Fig. 5f). We next examined the relative contribution of long and short ROS1 isoforms on invasive potential and EMT. When overexpressing cDNA constructs encoding either the long or short ROS1 isoform in NIH-3T3 cells^36^, we observed significantly higher invasiveness induced by the long isoform but not the short isoform (Fig. 5g and Supplementary Fig. 8).

We also tested if expression of wild-type versus S34F mutant U2AF1 in HCC78 cells altered the sensitivity of these cells to the ROS1 inhibitor crizotinib. We observed a modest but statistically significant difference in the IC50 levels for crizotinib (one-way ANOVA, *P* < 0.01 with *N* = 6; Fig. 5h), with U2AF1 S34F mutant cells showing higher sensitivity. Collectively, these observations suggest a functional interaction between U2AF1 S34F and ROS1 through alternative splicing, favoring expression of the long ROS1 isoform and subsequent increased invasive potential (Fig. 5i).

### Analysis of ROS1 isoforms in LUAD

Lastly, we attempted to examine the association of SLC34A2-ROS1 long and short isoforms with clinical outcome in TCGA LUAD cases. Unfortunately, the rarity of ROS1 fusion cases in TCGA (*n* = 11) prevented us from doing so. We separately explored if alternative splicing of the same exons in endogenous ROS1 was associated with outcome but found that in cases with quantifiable splicing at these positions (*n* = 90) there was no association with overall survival (*P* = 0.89).

## Discussion

When considering recurrent somatic alterations involving splicing factors found in human tumors, U2AF1 S34F mutations are unique in their specific enrichment within both a subset of myeloid malignancies and LUADs^35,56,57^. Our current study was motivated by our observation of significant co-association between U2AF1 S34F mutations and rearrangements of the ROS1 oncogene, which we extended to more than 2,000 genotyped LUADs. Fortuitously, one LUAD cell line (HCC78) harbors both mutations and thus allowed us to explore the interaction between the two mutations in cancer cells that acquired both during tumorigenesis. We confirmed the previously reported dependence of these cells on wild-type U2AF1^39^ using TALENs and therefore overexpressed epitope tagged versions of wild-type or S34F mutant U2AF1 in HCC78 cells. Using this system, we describe the genome-wide cross-linking interactions of mutant and wild-type U2AF1 proteins at nucleotide resolution by iCLIP. We also describe the impact of these interactions on RNA splicing, gene expression, cell growth and invasion phenotypes.

The S34F mutation is located within the first zinc-binding domain of U2AF, which a prior mutagenesis study showed is critical for binding to the 3′ splice site of pre-mRNAs. S34 is not one of the classically conserved zinc finger residues, suggesting that a substitution at this position could keep the zinc finger structure intact while potentially changing its binding properties. We were therefore initially surprised by our finding that there were no significant global changes in RNA binding properties of wild-type and mutant U2AF1. However, upon closer inspection, we found significant qualitative and quantitative differences between the two U2AF1 isoforms for binding within intronic 3′ splice sites. Specifically, we found that wild-type U2AF1 associates with ~86% of 3′ splice sites, similar to the previously described ~88% occupancy of the adjacent pyrimidine-rich region by the larger U2AF2 subunit when measured by CLIP-Seq^50^. In contrast, we observed ~10–20% diminished binding to 3′ splice sites by the S34F isoform, which suggests that the S34F mutation partially decreases the affinity of U2AF1 for 3′ splice sites. Furthermore, using the high resolution afforded by iCLIP, we were able to identify a single nucleotide shift in the cross-linking profiles of the wild-type and mutant U2AF1 proteins. This shift was accompanied by a change in 3′ splice site binding preference from TAG for the wild-type isoform to CAG for the S34F mutant isoform. These binding preferences matched the 3′ splice site sequences that were enriched in introns that we observed to be alternatively spliced in the presence of mutant U2AF1. Prior studies in human tumors and other cell lines observed a similar preference for CAG at exons involved in S34F-dependent alternative splicing events^38,39^. Thus, there is a direct link between binding changes induced by S34F and alternative splicing.

We also provide evidence for the co-association of U2AF1 S34F mutations and ROS1 fusions in LUADs. Given the relative rarity of both U2AF1 S34F and ROS1 translocations in LUAD, future larger studies will be required to confirm this association. That said, we identified an interaction between the two mutations in HCC78 cells. Specifically, we observed that the presence of S34F mutant U2AF1 results in differential splicing of short and long ROS1 isoforms and this was associated with increased invasive potential. Of note, not all ROS1 fusion proteins that have been reported in LUADs include exon 32 of ROS1, thus precluding formation of a long isoform similar to that observed in HCC78 cells. In such cases, a co-occurring U2AF1 S34F mutation could potentially still create other types of alternatively spliced variants. Additionally, it is possible U2AF1 S34F may still increase expression of genes involved in EMT in such cases.

The recent identification of recurrent mutations in splicing factors in diverse tumor types could have implications for cancer therapy^35,56,57^. While these mutations have been generally proposed as driver mutations^20,21,23,26,27,38,58^, such a functional role has yet to be convincingly determined in solid tumors. A previous study examining recurrent mutations in LUAD suggested a lower progression-free survival in patients harboring U2AF1 S34F mutations^20^, and the dependence on wild-type U2AF1 for carcinoma cell growth^39^. While U2AF1 is an essential gene required for cell survival, this finding suggests that there may be future opportunities for U2AF1-directed therapeutic targeting in lung cancers. For example, a recent study in MDS demonstrated therapeutic vulnerability of U2AF1-mutant cells to pharmacological modulation of the spliceosome by sudemycin D6^59^. Notably, similar to our observations on the role of mutant U2AF1 in LUADs, S34F mutations have also been found to induce phenotypic changes in hematopoiesis relevant to MDS and myeloid malignancies^60,61^. In LUAD cells we did not observe significant differences in the alternative splicing of the specific genes implicated in these prior studies, including ATG7, H2AFY, and STRAP, which were associated with transformation and an erythroid differentiation defect^60,61^.

The predilection of U2AF1 mutations for malignancies of specific tissue lineages and histological subtypes suggests context specificity for this gene′s role in oncogenesis, and the presence of cooperating factors in these tumors. While overlapping effects of U2AF1 mutations on gene expression and splicing have been observed in both myeloid neoplasms and lung carcinomas, the distribution of mutation hotspots are significantly different between them, as highlighted here. Separately, unlike a recent study showing the potentially dispensable role for U2AF1 mutations in inducible AML in mice^62^, multi-region sequencing of human lung cancers profiled in our study and a prior study^43^ revealed the universal presence of S34F lesions in distinct tumor deposits. Finally, the cooperativity that we observed between U2AF1 S34F and alternate splicing of ROS1 isoforms further supports the context specificity of these lesions.

In conclusion, in this study we report RNA binding specificities of wild-type and S34F mutant U2AF1. Additionally, we propose a potential functional role for U2AF1 S34F in LUADs. While our observations on the functional relationship between U2AF1 mutations and ROS1 fusions were made in HCC78 cells — the only cell line known to harbor both lesions — it is possible that they could generalize more broadly to LUADs harboring other driver mutations. Importantly, LUADs harboring ROS1 fusions can be highly responsive to tyrosine kinase inhibitors such as critzotinib^31^ and we observed a higher sensitivity to crizotinib in HCC78 cells overexpressing U2AF1 S34F versus wild-type. Future work should therefore address the impact of U2AF1 S34F mutations on the therapeutic activity of ROS1 kinase inhibitors or synergistic combinations in these tumors and the role of U2AF1 mutations in tumors without ROS1 fusions.

## Methods

### Human subjects

All biospecimens analyzed in this study were collected with informed consent from subjects enrolled on an Institutional Review Board-approved protocol at Stanford University. Comprehensive cancer genome profiling was performed using the Stanford Actionable Mutation Panel (STAMP) on 2100 tumor biopsy specimens from 1974 unique patients. Genotyping data were generated in the course of routine oncological clinical care at Stanford University, in a Clinical Laboratory Improvement Amendments (CLIA)–certified laboratory, between January 2015 and April 2018. De-identified data were aggregated from patients as profiled using two consecutive versions of the STAMP assay. STAMP v1 included exon 2 of U2AF1 (including the S34 hotspot) and STAMP v2 included exons 1–8. Base substitution and short insertions and deletions were examined to identify those likely to affect U2AF1 as well as 130 other recurrently mutated genes. In total, 20 distinct U2AF1 alterations were identified, comprised of 19 base substitutions and 1 in-frame insertion. These alterations displayed remarkably stereotyped sequence composition in U2AF1, with 9 hotspot mutations (S34F/Y) and 11 dispersed mutations, each occurring only once (Fig. 1a).

### Cell culture

All cells were cultured at 37 °C in a 5% CO_2_ incubator. HCC78 cells were cultured in RPMI-1640 (Thermo Fisher), supplemented with 10% fetal bovine serum (FBS), penicillin (100 U/ml) and streptomycin (100 µg/ml). NIH-3T3 and 293 T cells were cultured in DMEM (Lonza) supplemented with 10% FBS, penicillin (100 U/ml) and streptomycin (100 µg/ml).

### U2AF1 construct generation and expression

Wild-type or S34F mutant U2AF1 cDNA were generated using RNA from the HCC78 cell line harboring alleles. A Kozak consensus sequence and FLAG-6X HA tag were added to the N-terminus of U2AF1 isoforms. The resulting wild-type and mutant S34 FLAG-HA U2AF1 were cloned into a pTRIPz tet-inducible lentiviral vector, and used for transduction of HCC78 cells. Stable subclones were generated and validated by Sanger re-sequencing. Doxycycline was titrated in vitro to achieve expression levels of exogenous wild-type and S34F constructs similar to endogenous levels in HCC78, as confirmed by immunoblotting.

### ROS1 construct generation

The short and long ROS1 fusion constructs were graciously provided as a gift from Helen Zou (Pfizer), and the ROS1 constructs were expanded and selected according to the methods outlined by Zou and collegues^63^. Subsequent experiments utilizing these constructs are described in the quantitative-PCR and cell invasion assays.

### Lentivirus production

For lentivirus production, 293 T cells were used and cultured in DMEM (Lonza) + 10% FBS. 293 T cells were transfected with FuGENE HD (Promega), MD2.G (envelope, Addgene), psPAX2 (packaging, Addgene), and FLAG-HA-U2AF1 and FLAG-HA-U2AF1^S34F^ vectors, and Opti-MEM (Thermo Fisher) overnight. The viral supernatant was collected after 48 h, centrifuged at 1500 × *g* for 45 min, and filtered through a 0.45 µm membrane. The resulting supernatant was concentrated overnight at 4 °C following the Lenti-X concentrator protocol according to the manufacturer’s instructions (Clontech). The following day, the lentivirus was centrifuged at 1500 × *g* for 5 min, resuspended in PBS at 1/100^th^ the original volume and stored at −80 °C.

### iCLIP

FAST-iCLIP was performed on cells expressing Flag-HA-U2AF1_WT or Flag-HA-U2AF1_Mut. Cultured cells were UV crosslinked with a total of 0.35 J cm^−2^ ^47^. Whole-cell lysates were generated in iCLIP lysis buffer (50 mM HEPES, 200 mM NaCl, 1 mM EDTA, 10% glycerol, 0.1% NP-40, 0.2% Triton X-100, 0.5% N-lauroylsarcosine) and briefly sonicated using a probe-tip Branson sonicator to solubilize chromatin. Each sample was normalized for total protein amount, typically 0.5 mg, and partially digested with RNase A (ThermoFisher Scientific, EN0531) for 10 min at 37 °C and quenched on ice. Flag-HA-U2AF1 was isolated with anti-FLAG agarose beads (Sigma-Aldrich, A2220) for 1 h at 4 °C on rotation. Samples were washed sequentially in 1 mL for 5 min each at 4 °C: 2 × high stringency buffer (15 mM Tris-HCl, pH 7.5, 5 mM EDTA, 2.5 mM EGTA, 1% Triton X-100, 1% sodium deoxycholate, 120 mM NaCl, 25 mM KCl), 1 × high salt buffer (15 mM Tris-HCl pH 7.5, 5 mM EDTA, 2.5 mM EGTA, 1% Triton X-100, 1% sodium deoxycholate, 1 M NaCl), and 1 × NT2 buffer (50 mM Tris-HCl, pH 7.5, 150 mM NaCl, 1 mM MgCl2, 0.05% NP-40). Purified Flag-HA-U2AF1 was then eluted off anti-FLAG agarose beads using competitive FLAG peptide elution. Each sample was resuspended in 500 μl of FLAG elution buffer (50 mM Tris-HCl, pH 7.5, 250 mM NaCl, 0.5% NP-40, 0.1% sodium deoxycholate, 0.5 mg/ml FLAG peptide) and rotated at 4 °C for 30 min. The FLAG elution was repeated once for a total of 1 mL elution. Flag-HA-U2AF1 was then captured using anti-HA agarose beads (Pierce) for 1 h at 4 °C on rotation and samples were then washed as above.

After the NT2 wash, HA-bound RNA-protein complexes were dephosphorylated with T4 PNK (NEB, cat# M0210) for 30 min in an Eppendorf Thermomixer at 37 °C, 15 s 1500 g, 90 s rest in a 30 μL reaction, pH 6.5, containing 10 units of T4 PNK, 0.1 μL SUPERase-IN, and 6 μL of PEG-400 (16.7% final). After 30 min, beads were rinsed once with NT2 buffer and 3′-end ligated with T4 RNA Ligase 1 (NEB, M0204) overnight in an Eppendorf Thermomixer at 16 °C, 15 s 1500 g, 90 s rest in a 30 μL reaction containing 10 units T4 RNA Ligase 1, 1pmole pre-Adenylated-DNA-adapter, 0.1 μL SUPERase-IN, and 6 μL of PEG400 (16.7% final). The following day, samples were again rinsed with NT2 buffer and 5′ radiolabeled by adding 1 μL of T4 PNK, 0.5 μL g32-ATP (Perkin Elmer), 2 μL 10x T4 PNK Buffer, and 0.5 μL SUPERase-In, and 16 μL of water for 15 min at 37 °C. To this reaction, 1 μL of 100 mM DTT and 6 µL of 4× LDS Buffer (ThermoFisher Scientific) was added, samples heated to 75 °C for 10 min, and released RNA-protein complexes were separated on SDS–PAGE using NuPAGE 4–12% Bis-Tris Gels (1.0 mm × 12 well) at 180 V for 45 min. Resolved RNP complexes were wet-transferred to nitrocelluose at 400 mA for 60 min at 4 °C. RNA was recovered and processed for library preparation as in the irCLIP protocol^64^. Libraries, after PCR, were quantitated by HS-DNA Bioanalyzer (Agilent). Libraries were sequenced on an Illumina NextSeq machine using single-end 75-bp reads.

### iCLIP data analysis

FAST-iCLIP data were processed using the FAST-iCLIP analysis pipeline (https://github.com/ChangLab/FAST-iCLIP). PCR duplicates were removed using unique molecular identifiers (UMI) in the RT primer region. Adapter and barcode sequences were trimmed, and reads were mapped step-wise to repetitive and non-repetitive genomes. Specific parameters used are as follows: -f 14 (trims 13nt from the 5′ end of the read), -l 15 (includes all reads longer than 15nt), -bm 25 (minimum MAPQ score from bowtie2 of 25 is required for repeat element mapping), -sr 0.08 (STAR mismatch-per-base ratio; 0.08 corresponds to 2 mismatches per 25 bases), and -tr 2,2 (repetitive genome) and -tn 2,2 (nonrepetitive genome) RT stop intersection (*n*,*m*; where *n* = replicate number and *m* = number of unique RT stops required per n replicates). Using the -tr/tn 2,3 parameters, a minimum of 4 RT stops are required to support any single nucleotide identified as crosslinking site. The RT stops were ranked on a gene level by taking the mutant to wild-type ratio.

### 3′ SSs occupancy analysis

In order to evaluate occupancy of 3′ SSs, we considered any binding event within ±20 bps from a 3′ SS, and called that an “occupied” 3′ SS. We then calculated the total number of unique RT stops in a gene and finally defined the 3′ SS CLIP binding density as the average number of RT stops per 3′ SS for each gene. After sorting genes based on their absolute 3′ SS CLIP binding, we performed a “rolling quantile-0.75” with window size of 40 genes.

### Hexamer and tri-nucleotide analysis

Considering all hexamers, for *n* = 50 times, we uniformly sampled *M* 3′ SSs from all the annotated 3′ SSs and then calculated the mean and variance of the frequencies of all (*N* = 4^6^) possible hexamers. Denoting these by $\mu _{h_i},\sigma _{h_i}^2$ for all hexamers, *h*_i_,*i* = 1,…,*N*, this led to building a null hypothesis by which significance of the frequency of any given hexamer in our observed data was examined: For both U2AF1-WT and U2AF1-MUT, we first determined the factor *M*, which was set to the total number of unique 3′ SSs being occupied in each sample type, and then compared the frequencies of all hexamers, denoted by $f_{h_i}^{\mathrm{sample}}$. with that built for the null distribution, and calculated the *Z-*scores as follows:

$$\normalsize \normalsize z_{h_i}^{\mathrm{sample}} = \frac{{f_{h_i}^{\mathrm{sample}} - \mu _{h_i}}}{{\sigma _{h_i}}};i = 1, \ldots ,N;{\mathrm{sample}} \in U2AF1 - WT, U2AF1 - MUT.$$

These Z-scores were calculated in a sliding window of 5 bps around the inferred RT stops.

Similarly for all tri-nucleotides, we considered a window of size 3 from an inferred RT stop sliding over [*RT*−25,*RT*+25], where at each window, we calculated the frequency of each tri-nucleotide in U2AF1-MUT and compared that with that of U2AF1-WT. For this part, we used the Biostrings R package.

### RNA-Seq

Total RNA was extracted from cell lines using RNeasy Mini Kits (Qiagen). The isolated RNA was processed and prepared using the SMARTer^®^ Stranded Total RNA-Seq Kit (Clontech) according to the manufacturer’s protocol. Libraries were sequenced on Illumina NextSeq using paired-end 150-bp reads. In order to remove low-quality bases, we first optimized the trimming length using mapping rate and depth of coverage (data not shown). Then, the original 2×150 paired-end reads were trimmed to 2×100 reads. We then used STAR 2.3 to align the reads to the human genome transcriptome^65^. We used RSEM 1.2.29 package to calculate the gene-level FPKM values^66^. Differentially expressed genes were found by DESeq2 R package^67^. Next, in order to analyze pathway enrichment, we utilized the GSEA suit (in “weighted” Enrichment statistic mode) with the inferred coverage-based ranked set^68^ (ranked by fold change).

### Alternative splicing analysis

In order to find differentially spliced events in RNA-Seq data, we employed MISO^51^. We used a previously described database of known splicing events^32^. Insert length distribution and its statistics were inferred from the corresponding bam files, and were used for the MISO analysis. Five major categories of splicing events were used: alternative 3′ Splice Sites, alternative 5′ Splice Sites, *NAGNAG*, Exon Skipping, and Retained Intron. Associated with each annotated alternative splicing event MISO calculates the percent spliced in (PSI) measuring the inclusion of exon of interest. When comparing wild-type and mutant, we used two values to determine differentially spliced events: (1) difference in PSI, i.e. Δψ, and (2) Bayes factor which quantitates the significance of rejecting the “identical samples hypothesis”. As in previous studies, a Δψ = ±10% was applied to filter differentially spliced events^35^. Moreover, and in order to increase the specificity, we required a minimum Bayes factor of 5, and at least 10 supporting reads for an event to be called differentially spliced^35,51^.

### Trinucleotide preference in TCGA LUAD cases

In order to confirm the co-association between CAG or TAG sequences at 3′ splice sites and S34F mutation status we used a previously published PSI matrix for TCGA LUAD cases^69^. We first split the TCGA LUAD cohort into U2AF1 S34F mutant (group I) and wild-type (group II). We then defined the significantly alternatively spliced events as those with (1) significant *t*-test *p*-value in comparing two groups (less than 0.05) and (2) absolute difference of 2.5% or more in ψ values. We then extracted the differentially spliced exon(s) from the selected events and identified the sequences at their 3′ splice sites. A co-association test was then done by Fisher’s exact test for the number of exons with CAG/TAG (3 bases prior to their 3′ splice sites) when stratified by Δψ = ψ_groupI_−ψ_groupII_ >0.025 or ≤−0.025. Here, ψ_groupK_ is defined as the median ψ across all the samples in group K.

### Comparison of splicing in HCC78 and TCGA LUAD cases

In order to assess the relationship between differentially spliced events found in HCC78 and TCGA primary LUAD samples, we began with the PSI matrix described in the Trinucleotide preference in TCGA cases section. We used U2AF1 wild-type LUAD cases as controls and for each event overlapping with differentially spliced events found in HCC78 mutant vs wild-type, converted the ψ value into a Z-score, i.e. $Z_{i,j} = \frac{{{\mathrm{\Psi }}_{i,j} - \mu _{{\mathrm{\Psi }}_{{\mathrm{l}},{\mathrm{j}}};l \in WT_1,WT_2, \ldots }}}{{\sigma _{{\mathrm{\Psi }}_{l,j};l \in WT_1,WT_2, \ldots }}}$ where *i*and *j* correspond to event and sample, respectively. We then pooled all the events of all the cases, with Z-score greater than 1.64 (i.e. increased exon inclusion in S34F mutant; right tail 0.05 p-value) and increased inclusion (in HCC78 mutant) and counted the number of events. Similarly, we counted events with (Z >1.64 and decreased inclusion), (Z <−1.64 and increased inclusion), and (Z <−1.64 and decreased inclusion). From these, we built a contingency table and assessed the co-association via a Fisher’s exact test.

### Relationship between CLIP binding pattern and splicing

In order to evaluate the wild-type versus mutant differences in binding pattern in 3′ splice sites, we grouped 3′SS based on the strength of CLIP binding to their neighboring nucleotides. To do so, we defined deterministic rules based on the majority binding abundance. For any given 3′ SS, we first converted the binding amplitudes into a vector of fractions quantitating the strength of binding in the neighboring bases. We then considered a (limited) symmetrical window of 9 bps around the 3′SS, and sought to find the genomic position corresponding to the strongest binding fraction. Therefore, we defined 9 major “clusters”, where clusters *C*_1_ to *C*_9_ correspond to maximum binding at positions −6 to +2. Since it is likely that the maximum binding happens outside of our limited window, we defined one more cluster *C*_10_ to cover those 3′SSs. We further modified the major clusters by moving the 3′SS with maximum binding <10% in them to cluster *C*_10_. In order to find the effect of S34F mutation on the relationship between binding patterns from CLIP-Seq and the splicing from RNA-Seq, we evaluated the co-association between strongly differentially spliced events and those with *cluster switch* between wild-type and mutant. We define strongly differentially spliced events conservatively as those having Bayes factor of at least 10 while showing a Δψ >10% when comparing mutant to wild-type. In this analysis, we only considered major clusters defined above.

### Association of ROS1 isoforms with outcomes

For this analysis we defined a metric for endogenous ROS1 isoform expression using the PSI matrix of the TCGA LUAD cases from the SpliceSeq TCGA splicing database (https://bioinformatics.mdanderson.org/main/TCGASpliceSeq:Overview). We defined the ROS1 short versus long isoform as the events skipping or including exon 32, respectively. A total of 90 TCGA cases had quantifiable PSI values (i.e. non-NA). We then tested the association of overall survival with alternative splicing of exon 32 using a Cox proportional hazard model.

### Quantitative RNA Immunoprecipitation PCR

RNA immunoprecipitation was performed using the anti-U2AF35 antibody followed by Proteinase K (Qiagen) digestion at 65 °C for 15 min. Total RNA was purified and isolated using RNeasy Mini Kits (Qiagen). Synthesis and production of cDNA from 1 µg total RNA (quantitated by NanoDrop) was performed using the High Capacity cDNA Reverse Transcription Kit (Applied Biosystems, Foster City, CA). Quantitative PCR (qPCR) was performed on the ABI7900 Real-Time PCR System (Applied Biosystems, Life Technologies) utilizing SYBR® Green Master Mix (Thermo Fisher) and primers specific to the coding regions of the genes assessed in 3 technical replicates. The primers used for SLC34A2-ROS1 were: 5′ TCT TAG TAG CGC CTT CCA GCT G 3′ (SLC34A2 exon 4), 5′ TCT TCA GCT TTC TCC CAC TGT ATT G 3′ (ROS1 exon 32), and 5′ AGG TCA GTG GGA TTG TAA CAA CCA 3′ (ROS1 exon 34). Relative gene expression was determined by the ∆∆Ct method^70^.

### Immunoblotting

The following antibodies were used for immunoblotting in 1:1000 dilutions: anti-HA (Sigma H6908), anti-U2AF1 (abcam ab172614), anti-FLAG M2 (Sigma F3165); the anti-Histone H3 antibody (abcam ab1791) was used at 1:3000 dilution, and anti-ROS1 (Cell Signaling Technology, #3287); anti-E-cadherin (Cell Signaling Technology, #3195); anti-fibronectin (Proteintech., 15613-1) was used at 1:1000 dilution. To perform immunoblotting analysis, 0.03 and 0.06 µg/mL of doxycycline was added to a culture of HCC78 cells stably integrated with doxycycline-inducible FLAG-HA-U2AF1WT, FLAG-HA-U2AF1S34F. The cells were washed in PBS and lysed and scraped with RIPA buffer (Sigma) and HaltTM protease and phosphatase inhibitor cocktail (Thermo Fisher). To perform the Western blot analysis to analyze EMT markers and ROS1 expression, the HCC78 cells overexpressing U2AF1 or U2AF1-S34F were incubated with 0.1ug/ml of doxycycline for 1 or 5 days. Subsequently, the cells were harvested and prepared for Western blot analysis. Lysate samples were run on a 4–20% Mini PROTEAN® TGXTM Precast Protein Gel (Biorad) and followed by blotting with indicated antibodies, detected using infrared-conjugated secondary antibodies (IR Dye 800) and Odyssey Licor (Lincoln, NE, USA). The expression was determined by measuring band intensities using Image J software. The uncropped and unprocessed scans for Fig. 5f can be found in Source Data.

### Cell survival assay for HCC78 harboring U2AF1-S34F mutation

In total 10,000 cells were seeded into 96 well plate. After 24 h, the cells were exposed to crizotinib at the indicated concentration and time. Subsequently, the samples were incubated with 100 µl of CellTiter-Glo solution for 20 min at room temperature, and the cell survival rate was determined by analyzing luminescence with a Spectramax M3 microplate reader (Promega, Molecular Devices).

### Cell invasion assays

For cell invasion assays we employed soft-agar kits (Cell Biolabs, Inc, Corning) according to the manufacturer’s protocol. Briefly, 0.5 × 10^6^ cells were seeded into the upper chamber with serum-free DMEM media and the DMEM supplemented with 10% fetal bovine serum was added into lower chamber as a chemo-attractant. After 24–36 h, the invaded cells were lysed with lysis buffer containing CyQuant® GR dye for 1 h or counted by staining (Corning). Fluorescence was measured at an excitation wavelength of 480 nm and emission wavelength of 520 nm using a Spectramax spectrophotometer (Molecular Devices Sunnyvale, CA).

### Generation of isogenic HCC78 cell lines using TALENs

TALENs were designed with Cornell University’s web-based TAL Effector Nucleotide Targeter 2.0 to target U2AF1 Exon 1. They were assembled with Addgene’s Golden Gate TALEN and TAL Effector Kit 2.0. The workflow was adapted from Cermak et al.^69^. Donor vector design and backbone construct were graciously provided as a gift from Matthew Porteus, and the U2AF1 wild-type or S34F cDNA construct was inserted with a FLAG-His tag at the C terminus of the exogenous U2AF1. The nucleotide sequence of the exogenous U2AF1 Exon 1 was wobbled at every third base to prevent homologous recombination with the endogenous U2AF1 sequence, while still preserving amino acid sequence. Homologous recombination was performed by utilizing a pair of TALENs targeting the endogenous U2AF1 genomic DNA sequence at Exon 1 (underlined): cggcagcagtgtcgacggcagcggcggcggcgggtgggaaatggcggagt. The pair of TALEN constructs with the donor construct were then electroporated into HCC78 cell lines using the Neon Transfection System, grown for 2 weeks, single-cell sorted, and grown on 96-well plates in RPMI media as described above. The TALENs and donor vector transfected cells were cultured and cells were serially analyzed by flow cytometry every 72 h for GFP signal until there was consistent GFP positivity (2–4 weeks). The GFP positive cells were then single-cell sorted on a BD FACSAria and cultured in 96-well plates for 2–6 weeks before transferring to 12-well plates. Total DNA and RNA were isolated and analyzed for integration of U2AF1 wild-type and U2AF1 S34F donor constructs and expression using PCR amplification and Sanger Sequencing.

### Reporting summary

Further information on research design is available in the Nature Research Reporting Summary linked to this article.

## Supplementary information

Supplementary Information

Description of Additional Supplementary Files

Supplementary Data 1

Supplementary Data 2

Supplementary Data 3

Supplementary Data 4

Supplementary Data 5

Supplementary Data 6

Reporting Summary

## Data Availability

Sequence data used in this study have been deposited at the GEO database with accession number “GSE123989”.

## Source data

Source Data

## Code availability

Code used for the analyses is available upon request from the authors.
