# Supplementary material for: Functional significance of U2AF1 S34F mutations in lung adenocarcinomas
Source: Nat Commun. 2019 Dec 13;10:5712. doi: 10.1038/s41467-019-13392-y (PMC6911043; doi:10.1038/s41467-019-13392-y)
Supplement: Supplementary file 1 — Supplementary Information [file 41467_2019_13392_MOESM1_ESM.pdf]

Supplementary Information

Functional Significance of U2AF1 S34F Mutations in Lung Adenocarcinomas

Mohammad S. Esfahani[1,2,*], Luke J. Lee[1,*], Young-Jun Jeon[1,*], Ryan A. Flynn[3], Henning Stehr[1,4], Angela B. Hui[1], Noriko Ishisoko[5], Eric Kildebeck[6], Aaron M. Newman[7,8], Scott V. Bratman[7,§], Matthew H. Porteus[6], Howard Y. Chang[10], Ash A. Alizadeh[1,2,10,#], Maximilian Diehn[1,7,11#]

[1]Stanford Cancer Institute, Stanford University, Stanford, USA
[2]Division of Oncology, Department of Medicine, Stanford University, Stanford, USA
[3]Department of Chemistry, Stanford University, Stanford, USA
[4]Department of Pathology, Stanford University, Stanford, CA
[5]Department of Bioengineering, Stanford University, Stanford, USA
[6]Department of Pediatrics, Stanford University, Stanford, USA
[7]Institute for Stem Cell Biology and Regenerative Medicine, Stanford University, Stanford, USA
[8]Department of Biomedical Data Science, Stanford University, Stanford, USA
[9]Howard Hughes Medical Institute, Stanford University, Stanford, CA, USA
[10]Division of Hematology, Department of Medicine, Stanford University, Stanford, USA
[11]Department of Radiation Oncology, Stanford University, Stanford, USA

SI Guide

Supplementary Figure 1	Confirmation of U2AF1 S34F mutation and SLC34A2-ROS1 translocation in HCC78 cells, TALEN homologous recombination results, and quality control for U2AF1 CLIP experiments.
Supplementary Figure 2	Co-association of cross-linking cluster switching and significant alternative splicing.
Supplementary Figure 3	Sequence analysis of CLIP cross-linking peaks near splice junctions.
Supplementary Figure 4	Distribution of the frequency fold change of trinucleotides when measured by their cross-linking to mutant vs wild-type.
Supplementary Figure 5	Relative Expression of EMT Genes and relationship with cross-linking differences.
Supplementary Figure 6	Effect of wild-type and mutant U2AF1 overexpression on cell proliferation and effect of U2AF1 knockdown on the relative expression of SLC34A2-ROS1 isoforms.
Supplementary Figure 7	Relative expression of SLC34A2-ROS1 isoforms in HCC78 RNA-Seq data from Fei et al.
Supplementary Figure 8	Western blot of the ROS1 short and long isoforms with Histone H3 used as the loading control.

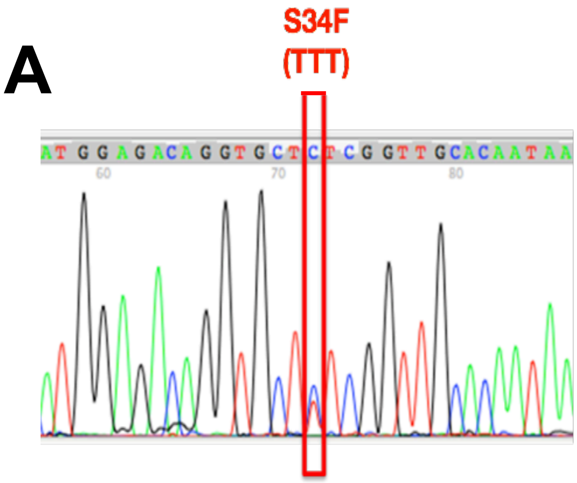

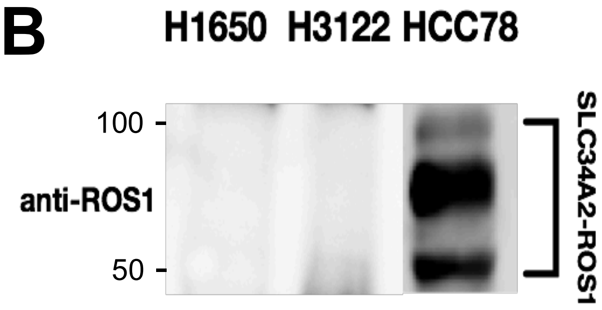

C

	TALEN Donor Wild-Type	TALEN Donor S34F
U2AF1 Wild-Type	6	0
U2AF1 S34F	0	3

p-value: 0.012

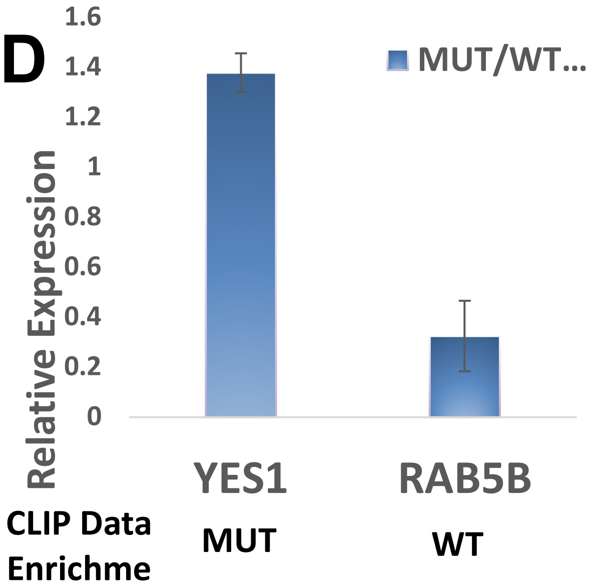

Supplementary Figure 1. Confirmation of U2AF1 S34F mutation and SLC34A2-ROS1 translocation in HCC78 cells, TALEN homologous recombination results, and quality control for U2AF1 CLIP experiments.

(A) Sanger sequencing of exon 2 of U2AF1 in HCC78 cells. Box indicates presence of heterozygous S34F mutation (TTT).

(B) Western blot with anti-ROS1 antibody detecting the presence of the SLC34A2-ROS1 fusion in HCC78 cells. Negative controls cell lines H1650 and H3122 are shown.

(C) Summary of homologous recombination events involving U2AF1 wild-type and S34F donor constructs in HCC78. TALEN, Transcription activator-like effector nuclease.

(D) RNA immunoprecipitation quantitative PCR (RIP-qPCR) validation of CLIP binding for wild-type and mutant U2AF1.

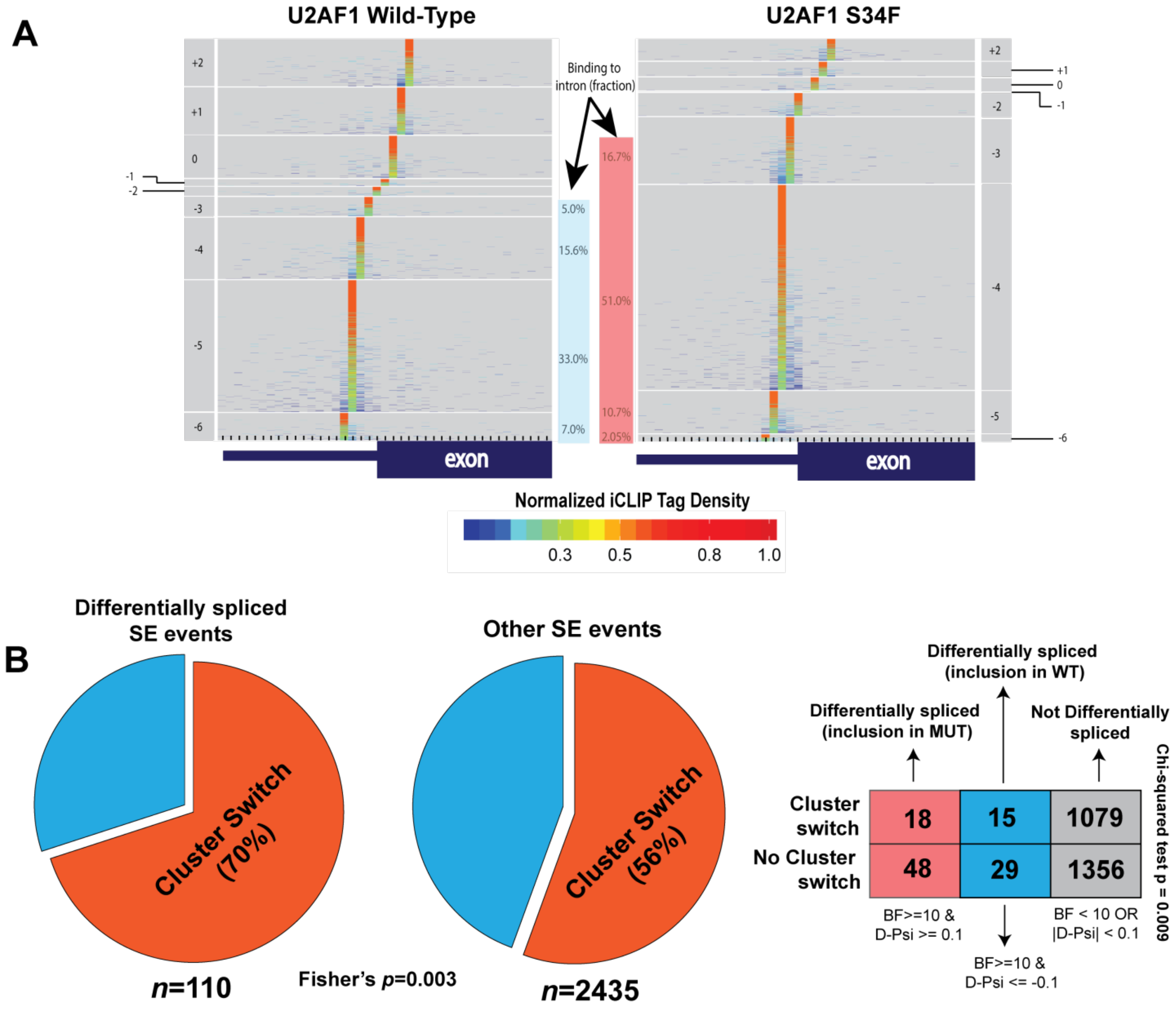

Supplementary Figure 2. Co-association of cross-linking cluster switching and significant alternative splicing.

(A) Heatmaps corresponding to wild-type and S34F mutant, showing the extent of binding to the neighboring positions next to the 3'SSs. Major binding occurs at position -5 and -4 for wild-type and mutant, respectively. Each row represents one coding 3' splice site.

(B) Differentially spliced events are defined as having $\Delta\Psi \geq 10\%$ and Bayes factor of at least 10. Cluster switch is defined as splice sites which appear in different clusters in wild-type versus mutant (excluding cluster 10). Differentially spliced skiped exon (SE) events between wild-type and mutant conditions were more likely to display cluster switching (P=0.003 by Fisher's exact test).

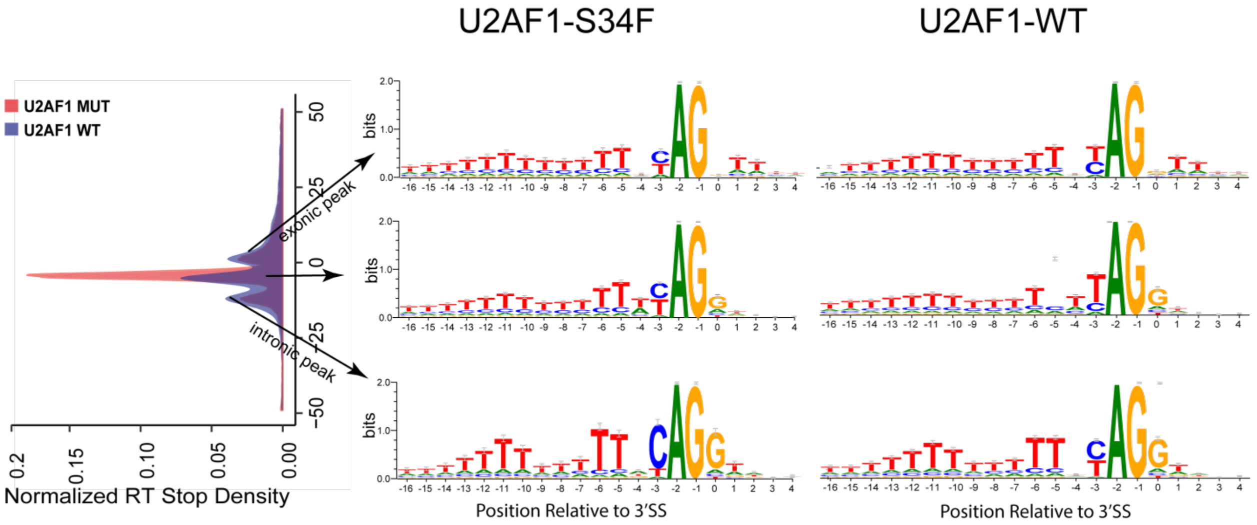

Supplementary Figure 3. Sequence analysis of CLIP cross-linking peaks near splice junctions.

Weblogo plots of the three cross-linking peaks near splice junctions observed in Fig. 3B. The three rows correspond to intronic (left-side peak), "main" (closer to 3' splice site from the intronic side) and exonic (right-side peak) peaks, respectively. The left and right columns correspond to mutant and wild-type, respectively. In all peaks, CAG is enriched in mutant compared to wild-type.

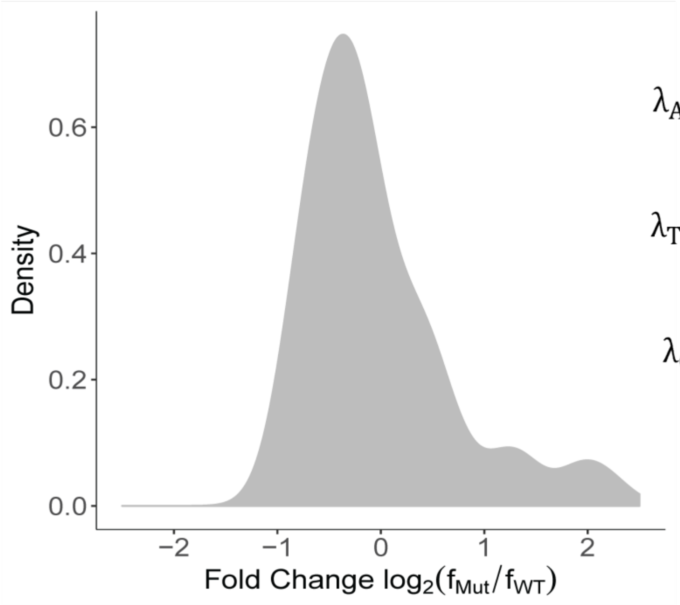

$$\lambda_{\mathrm{AAG}} = \operatorname{avg}\left[\log_2 \frac{f_{Mut,i}^{AAG}}{f_{WT,i}^{AAG}}\right]_{i\in\{-1,0,+1\}} = 0.3216$$

$$\lambda_{\mathrm{TAG}} = \operatorname{avg}\left[\log_2 \frac{f_{Mut,i}^{TAG}}{f_{WT,i}^{TAG}}\right]_{i\in\{-1,0,+1\}} = -0.833$$

$$\lambda_{\mathrm{CAG}} = \operatorname{avg}\left[\log_2 \frac{f_{Mut,i}^{CAG}}{f_{WT,i}^{CAG}}\right]_{i\in\{-1,0,+1\}} = 0.418$$

$$\kappa_{AAG:TAG} \coloneqq \lambda_{AAG} - \lambda_{TAG} = 1.15$$

Supplementary Figure 4. Distribution of the frequency fold change of trinucleotides when measured by their cross-linking to mutant vs wild-type.
The distribution of the scores, denoted by λ and defined as the average of mutant to wild-type fold change over a window of -/+1bp around the TSS (i denotes the relative distance from the RT site), corresponding to 64 possible trinucleotides. The three trinucleotides highlighted are: CAG (enriched in mutant), TAG (enriched in wild-type) and AAG (slightly in favor of mutant). Relative preference of AAG over TAG in mutant is 2.2-fold larger.

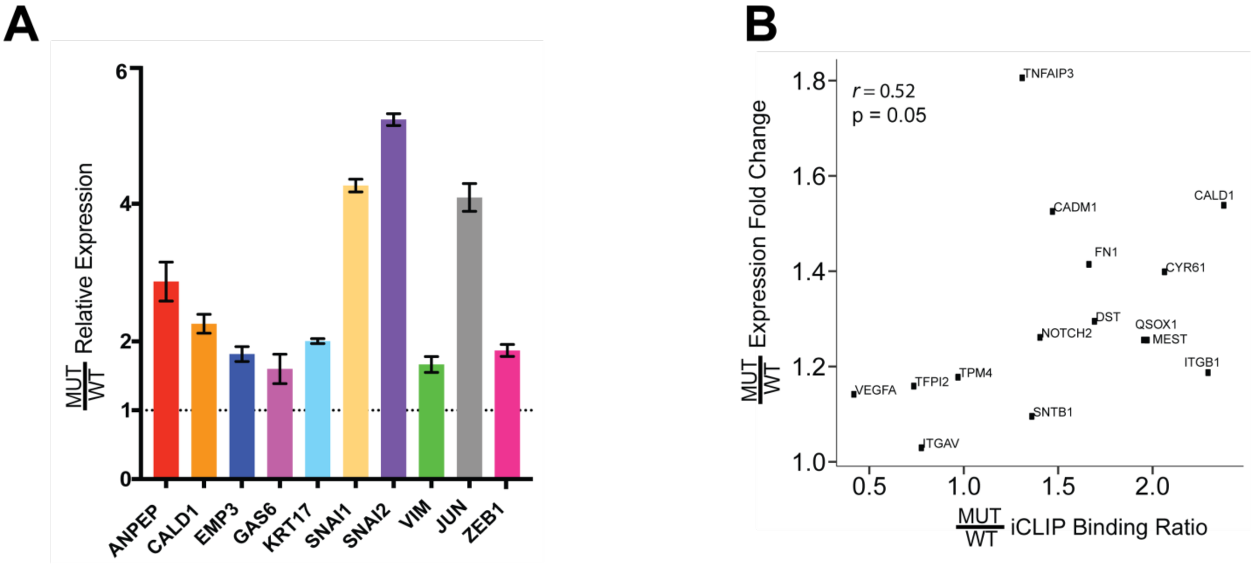

Supplementary Figure 5. Relative Expression of EMT Genes and relationship with cross-linking differences.

(A) Relative expression of EMT genes taking ratios of mutant to wild-type U2AF1 overexpression in HCC78. Experiments were performed in triplicate.

(B) Scatterplot of the EMT genes from the GSEA analysis that were present in both RNA-seq and iCLIP data sets. The Y-axis depicts the magnitude of differential expression between mutant and wild-type U2AF1 overexpression (in fold change); the X-axis shows the mutant to wild-type crosslinking ratio (in fold change; Spearman correlation of 0.52, P=0.05).

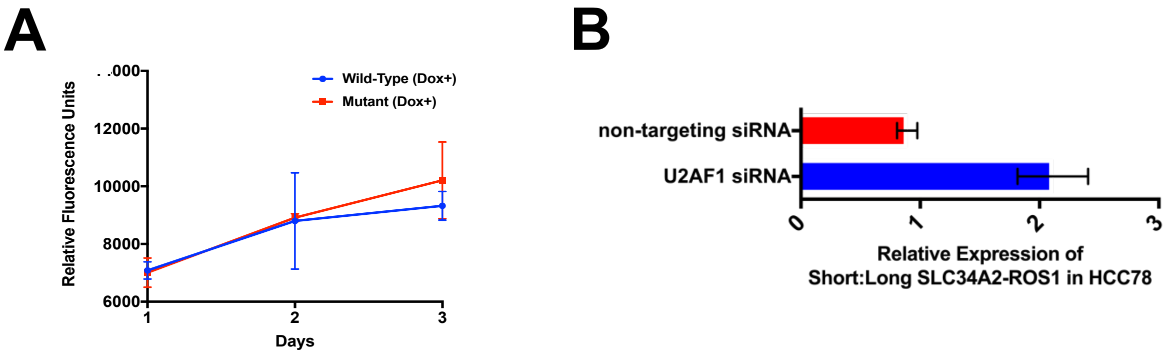

Supplementary Figure 6. Effect of wild-type and mutant U2AF1 overexpression on cell proliferation and effect of U2AF1 knockdown on the relative expression of SLC34A2-ROS1 isoforms.

(A) Cell growth assay after doxycycline-induced overexpression of wild-type and mutant U2AF1 measured by relative fluorescence units over 72 hours.

(B) siRNA knockdown of total U2AF1 using siRNA targeting the 3'UTR. Relative expression of short and long SLC34A2-ROS1 fusions were quantified by qPCR at exons 34 and 32, respectively. The error bars in both panels are used to show mean and standard deviations.

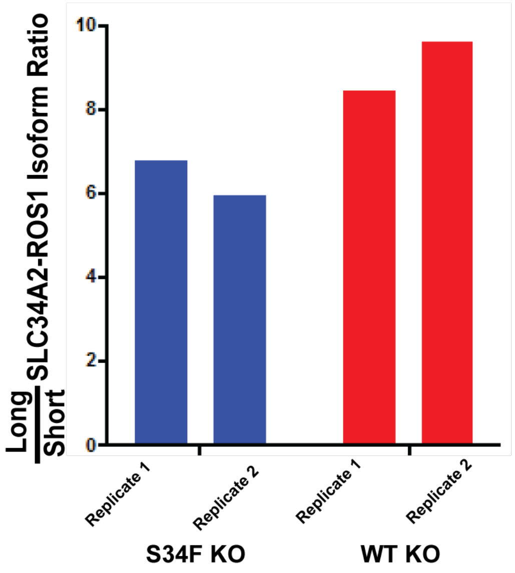

Supplementary Figure 7. Relative expression of SLC34A2-ROS1 isoforms in HCC78 RNA-Seq data from Fei et al.

Summary of long and short ROS1 fusion expression using data from Fei et al. "S34F KO" indicates cells with CRISPR-Cas9 knock-out using mutation-specific guide RNA; "WT KO" indicates use of control guide RNA against GFP.

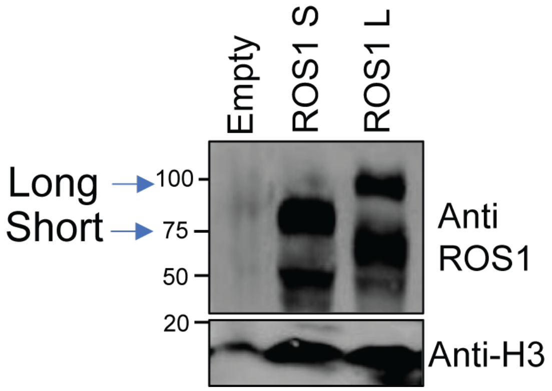

Supplementary Figure 8. Western blot of the ROS1 short and long isoforms with Histone H3 used as the loading control. The lowest molecular weight species seen below the long / short bands is a presumed degradation product of both ROS1 isoforms.
