# Supplementary material for: Functional significance of U2AF1 S34F mutations in lung adenocarcinomas
Source: Nat Commun. 2019 Dec 13;10:5712. doi: 10.1038/s41467-019-13392-y (PMC6911043; doi:10.1038/s41467-019-13392-y)
Supplement: Supplementary file 2 — Description of Additional Supplementary Files [file 41467_2019_13392_MOESM2_ESM.pdf]

Description of Additional Supplementary Files

File Name: Supplementary Data 1
Description: (a) Genotyping information for the two samples used in Figure 1B, and (b) ROS1 fusion partners in LUAD

File Name: Supplementary Data 2
Description: The genotyping of tumors with U2AF1-S34F and ROS1-wildtype

File Name: Supplementary Data 3
Description: iCLIP-seq- counts and enrichments for called peaks

File Name: Supplementary Data 4
Description: RNA-Seq FPKM Values- each row is one gene and columns are different conditions

File Name: Supplementary Data 5
Description: Alternative splicing PSI and Bayes factor data

File Name: Supplementary Data 6
Description: List of differentially spliced exons in EMT genes and their effect on making premature termination codons
