# Supplementary material for: Functional significance of U2AF1 S34F mutations in lung adenocarcinomas
Source: Nat Commun. 2019 Dec 13;10:5712. doi: 10.1038/s41467-019-13392-y (PMC6911043; doi:10.1038/s41467-019-13392-y)
Supplement: Supplementary file 9 — Reporting Summary [file 41467_2019_13392_MOESM9_ESM.pdf]

nature research

Corresponding author(s): Maximilian Diehn and Ash Alizadeh

Reporting Summary

Nature Research wishes to improve the reproducibility of the work that we publish. This form provides structure for consistency and transparency in reporting. For further information on Nature Research policies, see Authors & Referees and the Editorial Policy Checklist.

Statistical parameters

When statistical analyses are reported, confirm that the following items are present in the relevant location (e.g. figure legend, table legend, main text, or Methods section).

n/a	Confirmed	
☐	☒	The exact sample size (*n*) for each experimental group/condition, given as a discrete number and unit of measurement
☐	☒	An indication of whether measurements were taken from distinct samples or whether the same sample was measured repeatedly
☐	☒	The statistical test(s) used AND whether they are one- or two-sided<br>*Only common tests should be described solely by name; describe more complex techniques in the Methods section.*
☐	☒	A description of all covariates tested
☐	☒	A description of any assumptions or corrections, such as tests of normality and adjustment for multiple comparisons
☐	☒	A full description of the statistics including central tendency (e.g. means) or other basic estimates (e.g. regression coefficient) AND variation (e.g. standard deviation) or associated estimates of uncertainty (e.g. confidence intervals)
☐	☒	For null hypothesis testing, the test statistic (e.g. *F*, *t*, *r*) with confidence intervals, effect sizes, degrees of freedom and *P* value noted<br>*Give P values as exact values whenever suitable.*
☒	☐	For Bayesian analysis, information on the choice of priors and Markov chain Monte Carlo settings
☒	☐	For hierarchical and complex designs, identification of the appropriate level for tests and full reporting of outcomes
☒	☐	Estimates of effect sizes (e.g. Cohen's *d*, Pearson's *r*), indicating how they were calculated
☐	☒	Clearly defined error bars<br>*State explicitly what error bars represent (e.g. SD, SE, CI)*

Our web collection on statistics for biologists may be useful.

Software and code

Policy information about availability of computer code

Data collection	BioPortal and COSMIC were used to collect samples involving somatic point mutations in U2AF1. SQL was used to get inquiry from STAMP (Stanford Solid Tumor Actionable Mutation Panel) database.
Data analysis	All statistical tests were performed with R-3.3.2. R packages were installed from Bioconductor or CRAN-project. Raw fastq files of RNA-seq experiments were aligned using STAR, resulted in bam files. RNA-seq bam files were then analyzed by MISO for alternative splicing analysis (with Python 2.7.8), by GSEA v.2 for gene set enrichment analysis, by RSEM 1.2.29 for transcription quantification, and by DESeq.2 for differential expression analysis. In multiple places bedtools and samtools were used.

For manuscripts utilizing custom algorithms or software that are central to the research but not yet described in published literature, software must be made available to editors/reviewers upon request. We strongly encourage code deposition in a community repository (e.g. GitHub). See the Nature Research guidelines for submitting code & software for further information.

Data

Policy information about availability of data

All manuscripts must include a data availability statement. This statement should provide the following information, where applicable:

- Accession codes, unique identifiers, or web links for publicly available datasets
- A list of figures that have associated raw data
- A description of any restrictions on data availability

Sequencing data that support the findings of this study have been deposited in GEO with the accession codes GSE123989.

Field-specific reporting

Please select the best fit for your research. If you are not sure, read the appropriate sections before making your selection.

☒ Life sciences ☐ Behavioural & social sciences ☐ Ecological, evolutionary & environmental sciences

For a reference copy of the document with all sections, see nature.com/authors/policies/ReportingSummary-flat.pdf

Life sciences study design

All studies must disclose on these points even when the disclosure is negative.

Sample size	A statistician was intimately involved in experimental design and analysis, although sample size was not always able to be predetermined. All experiments were performed with 2-4 replicates, as detailed below. Follow up experiments were performed at least three times. Experiments were repeated and statistical analyses performed as described to test for significance.
Data exclusions	No data were selectively excluded.
Replication	All experiments were done in triplicate or quadruplicate with the exception of CLIP-Seq, which due sample limitations and the experimental design was done in duplicate. The attempts at replication where relevant showed the same conclusions and the representative data are shown in the manuscript.
Randomization	N/A
Blinding	N/A

Reporting for specific materials, systems and methods

Materials & experimental systems

n/a	Involved in the study	
☐	☒	Unique biological materials
☐	☒	Antibodies
☐	☒	Eukaryotic cell lines
☒	☐	Palaeontology
☒	☐	Animals and other organisms
☐	☒	Human research participants

Methods

n/a	Involved in the study	
☒	☐	ChIP-seq
☒	☐	Flow cytometry
☒	☐	MRI-based neuroimaging

Unique biological materials

Policy information about availability of materials

Obtaining unique materials	The cell lines generated in this study are available by request from the authors.

Antibodies

Antibodies used	anti-HA (Sigma H6908), anti-U2AF35 (abcam ab172614), anti-FLAG M2 (Sigma F3165); the anti-Histone H3 antibody (abcam ab1791), ROS1 (Cell Signaling 3287), anti-E-cadherin (Cell Signaling Technology, #3195), anti-fibronectin (Proteintech., 15613-1)

Validation	All antibodies were commercial and validated by the manufacturers. We used expected migration sizes for the proteins and tags of interest to separately confirm the specificity using immunoblots.

Eukaryotic cell lines

Policy information about cell lines

Cell line source(s)	HCC-78 derived from ATCC.
Authentication	The cell lines were authenticated by testing for the SLC34A2-ROS1 fusion junctional sequence using PCR.
Mycoplasma contamination	The cell lines were tested for mycoplasma contamination and were negative.
Commonly misidentified lines (See ICLAC register)	None.

Human research participants

Policy information about studies involving human research participants

Population characteristics	Comprehensive cancer genome profiling was performed using the Stanford Actionable Mutation Panel (STAMP) on 2100 tumor biopsy specimens from 1974 unique patients. Genotyping data were generated in the course of routine oncological clinical care at Stanford University, in a Clinical Laboratory Improvement Amendments (CLIA)–certified laboratory, between January 2015 and April 2018. De-identified data were aggregated from patients as profiled using two consecutive versions of the STAMP assay.
Recruitment	No recruitment was done specifically for this study
