# Supplementary material for: Functional significance of U2AF1 S34F mutations in lung adenocarcinomas
Source: Nat Commun. 2019 Dec 13;10:5712. doi: 10.1038/s41467-019-13392-y (PMC6911043; doi:10.1038/s41467-019-13392-y)

Related to Figure 5B

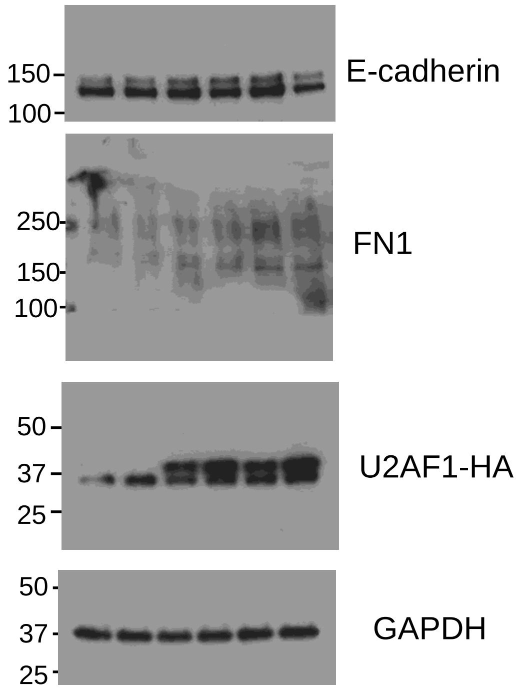

Related to Figure 5F

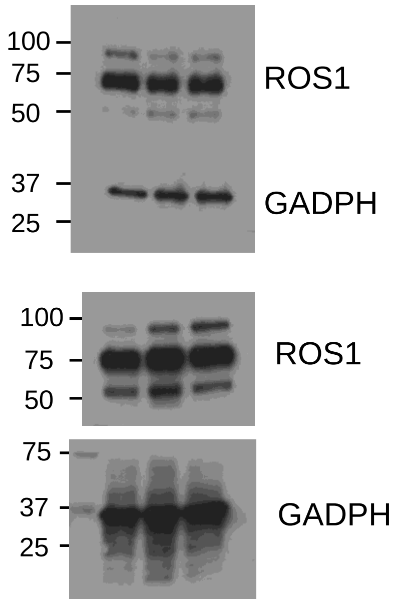

Uncropped and unprocessed scan: ROS1 in U2AF1-WT

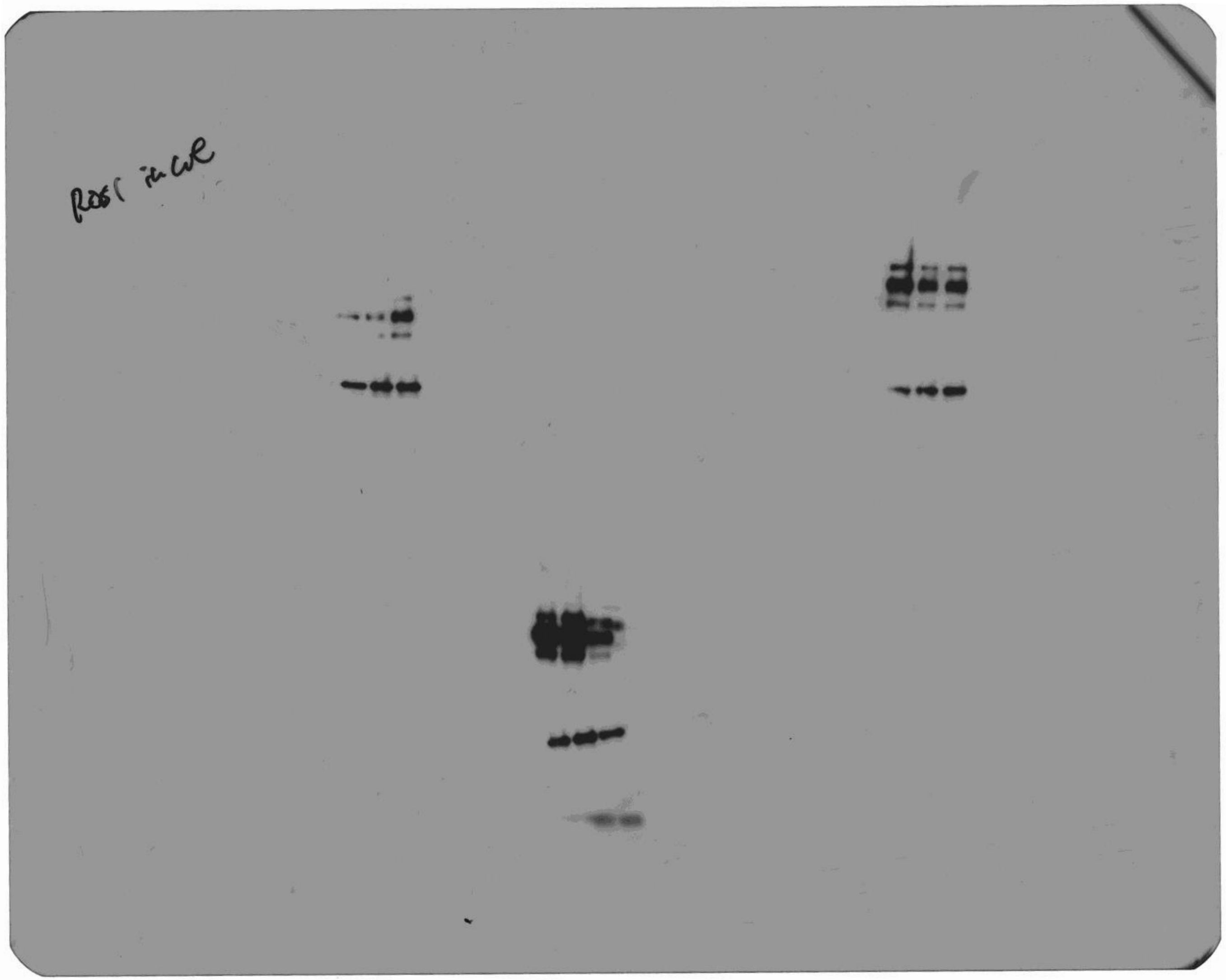

Uncropped and unprocessed scan: ROS1 in U2AF1-S34F

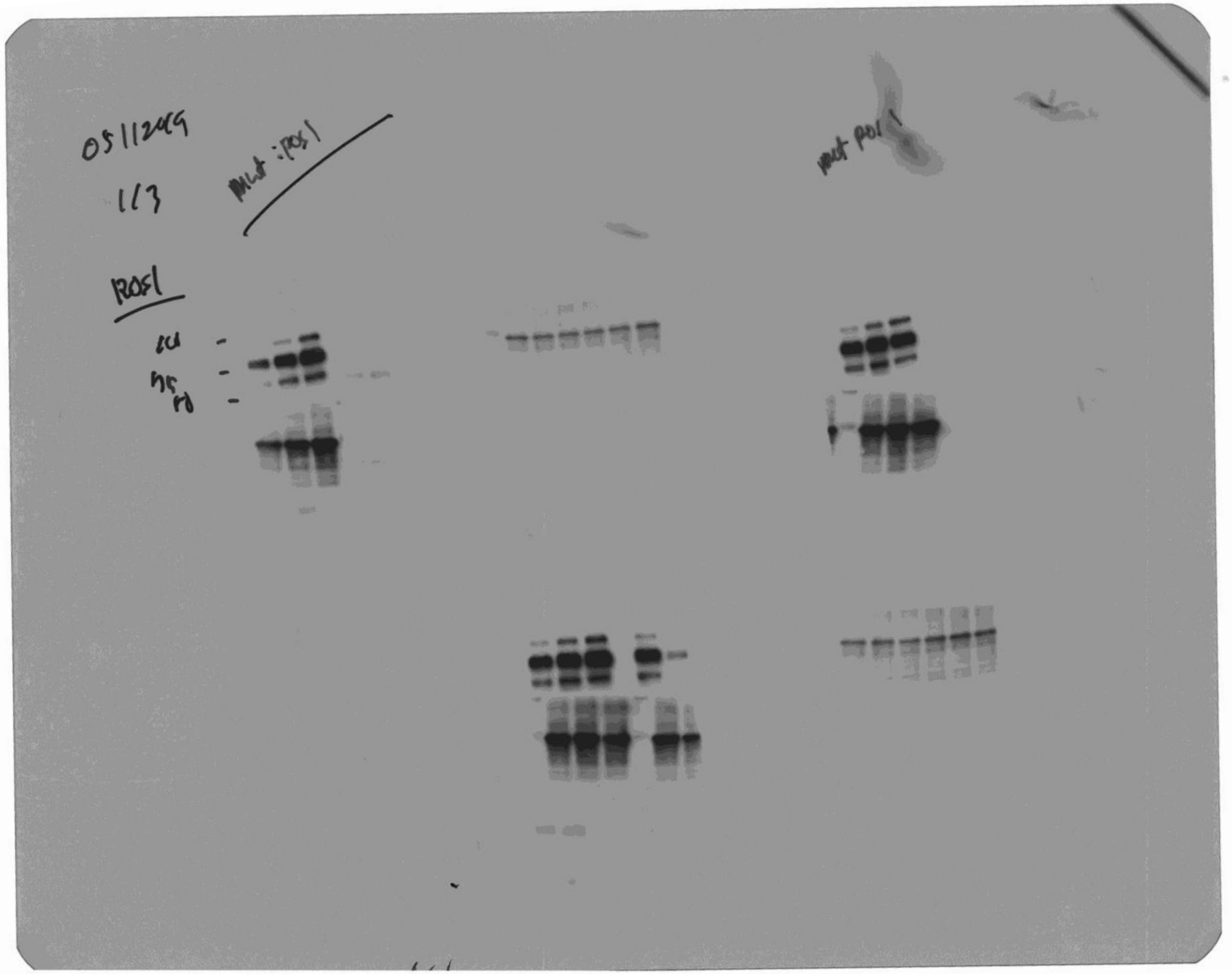

Supplement: Supplementary file 10 — Source Data [file 41467_2019_13392_MOESM10_ESM.pdf]
